# Reassortment of Human and Animal Rotavirus Gene Segments in Emerging DS-1-Like G1P[8] Rotavirus Strains

**DOI:** 10.1371/journal.pone.0148416

**Published:** 2016-02-04

**Authors:** Satoshi Komoto, Ratana Tacharoenmuang, Ratigorn Guntapong, Tomihiko Ide, Takao Tsuji, Tetsushi Yoshikawa, Piyanit Tharmaphornpilas, Somchai Sangkitporn, Koki Taniguchi

**Affiliations:** 1 Department of Virology and Parasitology, Fujita Health University School of Medicine, Toyoake, Aichi, Japan; 2 Department of Medical Sciences, National Institute of Health, Nonthaburi, Thailand; 3 Department of Microbiology, Fujita Health University School of Medicine, Toyoake, Aichi, Japan; 4 Department of Pediatrics, Fujita Health University School of Medicine, Toyoake, Aichi, Japan; 5 Department of Disease Control, Ministry of Public Health, Nonthaburi, Thailand; University of Hong Kong, HONG KONG

## Abstract

The emergence and rapid spread of novel DS-1-like G1P[8] human rotaviruses in Japan were recently reported. More recently, such intergenogroup reassortant strains were identified in Thailand, implying the ongoing spread of unusual rotavirus strains in Asia. During rotavirus surveillance in Thailand, three DS-1-like intergenogroup reassortant strains having G3P[8] (RVA/Human-wt/THA/SKT-281/2013/G3P[8] and RVA/Human-wt/THA/SKT-289/2013/G3P[8]) and G2P[8] (RVA/Human-wt/THA/LS-04/2013/G2P[8]) genotypes were identified in fecal samples from hospitalized children with acute gastroenteritis. In this study, we sequenced and characterized the complete genomes of strains SKT-281, SKT-289, and LS-04. On whole genomic analysis, all three strains exhibited unique genotype constellations including both genogroup 1 and 2 genes: G3-P[8]-I2-R2-C2-M2-A2-N2-T2-E2-H2 for strains SKT-281 and SKT-289, and G2-P[8]-I2-R2-C2-M2-A2-N2-T2-E2-H2 for strain LS-04. Except for the G genotype, the unique genotype constellation of the three strains (P[8]-I2-R2-C2-M2-A2-N2-T2-E2-H2) is commonly shared with DS-1-like G1P[8] strains. On phylogenetic analysis, nine of the 11 genes of strains SKT-281 and SKT-289 (VP4, VP6, VP1-3, NSP1-3, and NSP5) appeared to have originated from DS-1-like G1P[8] strains, while the remaining VP7 and NSP4 genes appeared to be of equine and bovine origin, respectively. Thus, strains SKT-281 and SKT-289 appeared to be reassortant strains as to DS-1-like G1P[8], animal-derived human, and/or animal rotaviruses. On the other hand, seven of the 11 genes of strain LS-04 (VP7, VP6, VP1, VP3, and NSP3-5) appeared to have originated from locally circulating DS-1-like G2P[4] human rotaviruses, while three genes (VP4, VP2, and NSP1) were assumed to be derived from DS-1-like G1P[8] strains. Notably, the remaining NSP2 gene of strain LS-04 appeared to be of bovine origin. Thus, strain LS-04 was assumed to be a multiple reassortment strain as to DS-1-like G1P[8], locally circulating DS-1-like G2P[4], bovine-like human, and/or bovine rotaviruses. Overall, the great genomic diversity among the DS-1-like G1P[8] strains seemed to have been generated through reassortment involving human and animal strains. To our knowledge, this is the first report on whole genome-based characterization of DS-1-like intergenogroup reassortant strains having G3P[8] and G2P[8] genotypes that have emerged in Thailand. Our observations will provide important insights into the evolutionary dynamics of emerging DS-1-like G1P[8] strains and related reassortant ones.

## Introduction

Group A rotavirus (RVA), a member of the *Reoviridae* family, is the primary pathogen causing acute gastroenteritis in the young of humans and many animal species worldwide. In humans, RVA infections are associated with high morbidity and mortality, being responsible for an estimated 453,000 deaths among children under 5 years of age annually [1]. More than half of these deaths are estimated to occur in developing countries in Asia and Africa [1–3]. The mature RVA particle is a triple-layered, non-enveloped icosahedron enclosing an 11-segment genome of double-stranded (ds)RNA [4]. The segmented nature of the genome enables reassortment between/within human and animal strains, and reassortment plays one of the major roles in the generation of the genomic diversity of this medically important virus [5].

RVA has two outer capsid proteins, VP7 and VP4, which independently elicit neutralizing antibody responses, and define the G and P genotypes, respectively [3, 5]. At least 27 G and 37 P genotypes have been recognized to date for RVAs [6, 7]. In human RVAs, 6 G genotypes (G1-4, G9, and G12) and 3 P genotypes (P[4], P[6], and P[8]) are commonly associated with human infections [5, 8, 9].

A whole genome-based genotyping system was recently proposed for RVAs based on the assignment to all the 11 gene segments (i.e., G/P and non-G/P defining genes) [10]. In this genotyping system, the acronym Gx-P[x]-Ix-Rx-Cx-Mx-Ax-Nx-Tx-Ex-Hx, where x is an integer, defines the genotype of the VP7-VP4-VP6-VP1-VP2-VP3-NSP1-NSP2-NSP3-NSP4-NSP5 genes, respectively, of a given RVA strain [3, 5]. The majority of human RVAs possess genes similar in sequence to those of prototype human strain Wa (genogroup 1 genes) or DS-1 (genogroup 2 genes) [11]. The Wa-like strains are characterized by non-G/P genotypes comprising I1-R1-C1-M1-A1-N1-T1-E1-H1, and tend to have G/P genotypes G1P[8], G3P[8], G4P[8], G9P[8], G12P[6], and G12P[8], whereas the DS-1-like strains are characterized by non-G/P genotypes comprising I2-R2-C2-M2-A2-N2-T2-E2-H2, and tend to have G/P genotypes G2P[4] [3, 5]. Although intergenogroup reassortants can exist, it is believed that such RVAs having both genogroup 1 and 2 genes exhibit decreased evolutionary fitness compared to that of the parental strains and thus would be selected against in nature [11, 12]. However, the emergence of unusual human intergenogroup reassortant strains (DS-1-like G1P[8] strains) having DS-1-like non-G/P genotypes (I2-R2-C2-M2-A2-N2-T2-E2-H2) together with G/P genotypes G1P[8]: G1-P[8]-I2-R2-C2-M2-A2-N2-T2-E2-H2, which had never been previously described, were recently reported in multiple districts in Japan and Thailand, Asia [13–15]. Notably, DS-1-like G1P[8] strains have become widespread in the human population and are predominant in several separate locations in Japan.

The first DS-1-like G1P[8] strains, including strains HC12016, NT004, and OH3506, were detected in children with severe gastroenteritis in Japan in 2012 [13–15], and subsequently, such unusual intergenogroup reassortant strains (PCB-180, SKT-109, and SSKT-41) were identified in Thailand in 2013 [16]. In 2013, we detected three DS-1-like intergenogroup reassortant strains having G3P[8] and G2P[8] genotypes, strains SKT-281, SKT-289, and LS-04, in diarrheic children in Phechaboon and Sukhothai Provinces, Thailand, a total of 687 RVA-positive stool samples being examined by PCR-based G and P genotyping, and polyacrylamide gel electrophoresis (PAGE) analysis (Tacharoenmuang et al., in preparation). To our knowledge, strains SKT-281, SKT-289, and LS-04 are the first DS-1-like intergenogroup reassortant strains having G3P[8] and G2P[8] genotypes that have emerged in Thailand.

Whole genome-based analysis is a reliable method for obtaining precise information on the origin of a given RVA strain, and for tracing its evolutionary pattern [10, 17]. To date, the full genome sequences of several DS-1-like G1P[8] strains from Japan and Thailand have been determined and characterized, which indicated the occurrence of reassortment event(s) between Wa-like G1P[8] and DS-1-like G2P[4] human RVAs [13, 15, 16]. Furthermore, DS-1-like G1P[8] strains in Japan and Thailand are very closely related to one another in all the 11 genes, showing the derivation of these strains from a common origin [16]. However, as the exact evolutionary pattern of DS-1-like G1P[8] strains remains to be elucidated, whole genomic analysis of the DS-1-like intergenogroup reassortant strains having G3P[8] and G2P[8] genotypes, strains SKT-281, SKT-289, and LS-04, might be useful for obtaining a more precise understanding of the evolutionary pattern of DS-1-like G1P[8] strains and related reassortant ones. In this study, deep sequencing using the next generation sequencing (NGS) Illumina MiSeq platform was performed to determine the complete nucleotide sequences of the whole genomes of these three DS-1-like intergenogroup reassortant strains. Furthermore, the whole genomes of six locally circulating human RVAs (two Wa-like G1P[8] and four DS-1-like G2P[4] strains) were also sequenced as references.

## Materials and Methods

### Ethics statement

The study was approved by the Ethical Review Committee for Research on Human Subjects of the Ministry of Public Health, Thailand (Ref. no. 10/2555). In this study, written informed consent for the testing of stool samples for RVAs and characterization of identified RVA strains was obtained from the children’s parents/guardians.

### Virus strains

The full-genomic sequences were determined for strains SKT-281, SKT-289, and LS-04, and locally circulating strains PCB-118, SKT-98, BD-20, NP-M51, SKT-138, and SSKT-133, which were identified in nine stool samples from hospitalized children aged 16–51 months with severe diarrhea in Phechaboon and Sukhothai Provinces, Thailand, during the RVA surveillance program in those provinces in 2012–2014, which involved a total of 3002 fecal specimens (Tacharoenmuang et al., in preparation). Of the 3002 fecal samples, RVA infection was detected in 687 (22.9%). Stool specimens were collected during hospital-based surveillance activities during the RVA vaccine effectiveness evaluation study in Thailand. The stool samples were examined by PCR-based G and P genotyping, and PAGE analysis at the National Institute of Health, Thailand. For Illumina MiSeq sequencing, the samples were submitted to Fujita Health University, Japan. Fecal specimens containing strains SKT-281, SKT-289, LS-04, PCB-118, SKT-98, BD-20, NP-M51, SKT-138, and SSKT-133 were kept at −30°C until use.

### Viral dsRNA extraction

Extraction of viral dsRNAs was performed as described previously [3, 18, 19]. Briefly, the viral dsRNAs were extracted from stool suspensions using a QIAamp Viral RNA Mini Kit (Qiagen). The extracted dsRNAs were used for (i) PAGE analysis and (ii) whole genomic analysis. For PAGE analysis, the dsRNAs were electrophoresed in a 10% polyacrylamide gel for 16 h at 20 mA at room temperature, followed by silver staining [20] to determine the genomic dsRNA profiles. For whole genomic analysis, viral dsRNAs were subjected to Illumina MiSeq sequencing as described below.

### cDNA library building and Illumina MiSeq sequencing

Preparation of a cDNA library and Illumina MiSeq sequencing were carried out as described previously [3, 18, 19]. Briefly, a 200 bp fragment library ligated with bar-coded adapters was constructed for strains SKT-281, SKT-289, LS-04, PCB-118, SKT-98, BD-20, NP-M51, SKT-138, and SSKT-133 using an NEBNext Ultra RNA Library Prep Kit for Illumina v1.2 (New England Biolabs) and an NEBNext Multiplex Oligos for Illumina (Index Primers Sets 1 and 2) (New England Biolabs) according to the manufacturer’s instructions. Library purification was performed using Agencourt AMPure XP magnetic beads (Beckman Coulter). The quality of the purified cDNA library was assessed on an Agilent 2100 Bioanalyzer (Agilent Technologies). Nucleotide sequencing was carried out on an Illumina MiSeq sequencer (Illumina) using a MiSeq Reagent Kit v2 (Illumina) to generate 151 paired-end reads. Data analysis was carried out using CLC Genomics Workbench v8.0.1 (CLC Bio). Contigs were assembled from the obtained sequence reads by *de novo* assembly. Using the assembled contigs as query sequences, the Basic Local Alignment Search Tool (BLAST) non-redundant nucleotide database was searched to obtain the full-length nucleotide sequence of each gene segment of strains SKT-281, SKT-289, LS-04, PCB-118, SKT-98, BD-20, NP-M51, SKT-138, and SSKT-133. The nucleotide sequences were translated into amino acid sequences using GENETYX v11 (GENETYX).

### Determination of RVA genotypes

The genotype of each of the 11 gene segments of strains SKT-281, SKT-289, LS-04, PCB-118, SKT-98, BD-20, NP-M51, SKT-138, and SSKT-133 was determined using the RotaC v2.0 automated genotyping tool (http://rotac.regatools.be/) [21] according to the guidelines proposed by the Rotavirus Classification Working Group (RCWG).

### Phylogenetic analyses

Sequence comparisons were carried out as described previously [3, 18, 19]. Briefly, multiple alignment of each viral gene was carried out using CLUSTAL W [22]. Phylogenetic trees were constructed using the maximum likelihood method and the Tamura-Nei substitution model using MEGA6.06 [23]. The reliability of the branching order was estimated from 1000 bootstrap replicates [24]. The results of phylogenetic analyses were validated using several other genetic distance models, such as the Kimura 2-parameter, Tamura 3-parameter, Jukes-Cantor, and Hasegawa-Kishino-Yano ones (data not shown).

### Nucleotide sequence accession numbers

The nucleotide sequence data presented in this manuscript have been deposited in the DDBJ and EMBL/GenBank data libraries. The accession numbers for the nucleotide sequences of the VP1-4, VP6-7, and NSP1-5 genes of strains SKT-281, SKT-289, LS-04, PCB-118, SKT-98, BD-20, NP-M51, SKT-138, and SSKT-133 are LC086714-LC086724, LC086725-LC086735, LC086736-LC086746, LC086747-LC086757, LC086758-LC086768, LC086769-LC086779, LC086780-LC086790, LC086791-LC086801, and LC086802-LC086812, respectively.

## Results and Discussion

### Profiles of genomic dsRNAs of strains SKT-281, SKT-289, and LS-04 on PAGE

**Fig 1:**
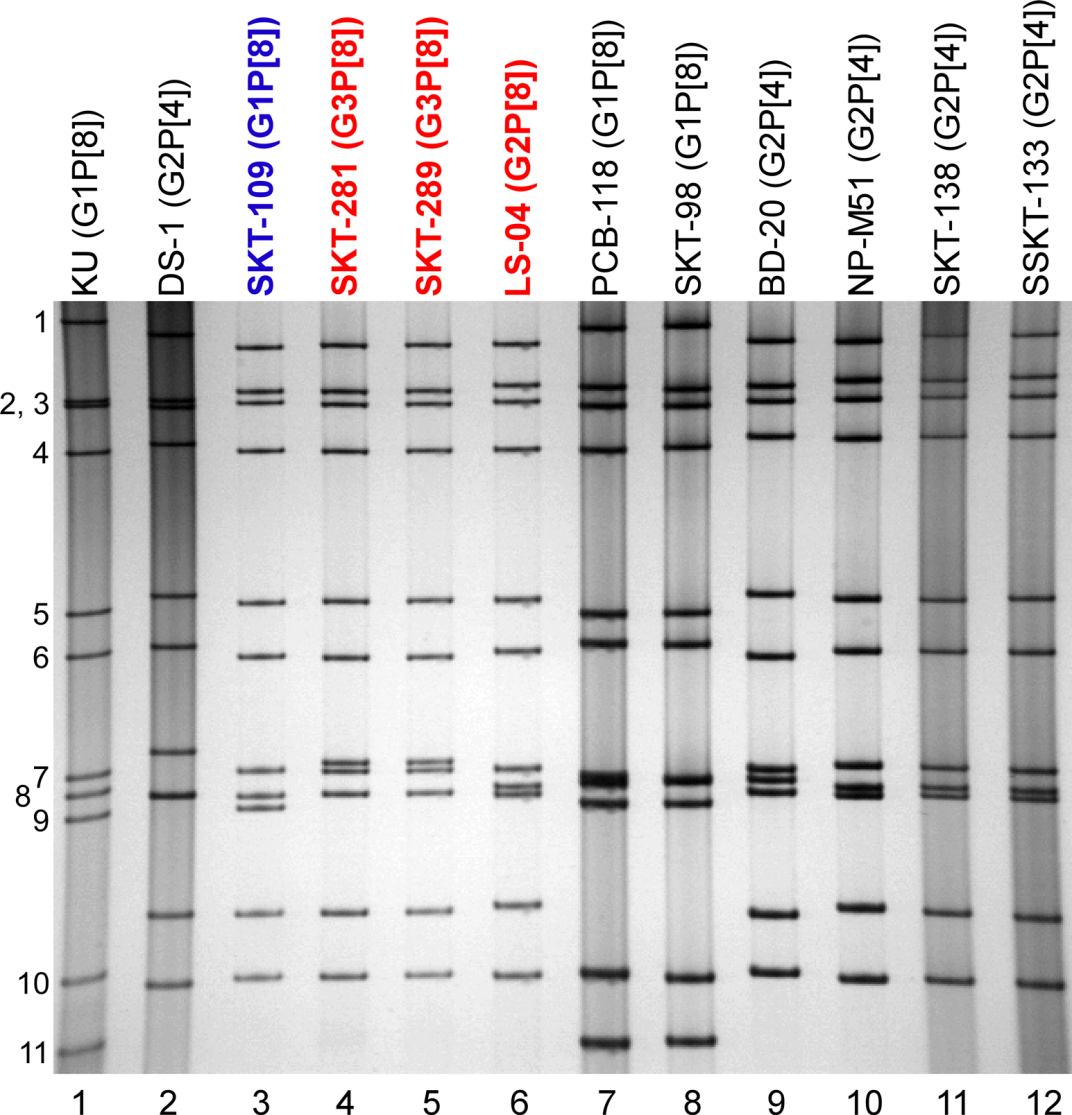
Genomic dsRNA profiles of strains SKT-281, SKT-289, and LS-04. Strains SKT-281, SKT-289, and LS-04 are shown in red, while DS-1-like G1P[8] strain SKT-109 is shown in blue. Lanes 1–2, dsRNAs of strains KU (G1P[8]) (lane 1) and DS-1 (G2P[4]) (lane 2) extracted from the cell cultures; lanes 3–6, dsRNAs of strains SKT-109 (lane 3), SKT-281 (lane 4), SKT-289 (lane 5), and LS-04 (lane 6) extracted from stool samples; and lanes 7–12, dsRNAs of co-circulating strains PCB-118 (lane 7), SKT-98 (lane 8), BD-20 (lane 9), NP-M51 (lane 10), SKT-138 (lane 11), and SSKT-133 (lane 12) extracted from fecal specimens. The numbers on the left indicate the order of the genomic dsRNA segments of strain KU.

The virion dsRNAs of strains SKT-281, SKT-289, and LS-04 were extracted from stool specimens and then analyzed by PAGE. Fig 1 shows the profiles of viral dsRNAs from strains SKT-281 (lane 4), SKT-289 (lane 5), and LS-04 (lane 6) from stool samples. Differing from the long electropherotype found in most Wa-like strains, strains SKT-281, SKT-289, and LS-04 showed the short electropherotype found in most DS-1-like strains. Of note was that strains SKT-281 and SKT-289 had an almost identical electropherotype, suggesting a close genetic relatedness between the two strains.

### Nucleotide sequencing and whole-genome-based genotyping of strains SKT-281, SKT-289, and LS-04

In order to gain an insight into the genetic variability among strains SKT-281, SKT-289, and LS-04, and their genetic relatedness with other RVA strains worldwide, the full-genome sequences of all the 11 segments of these three strains were determined using the NGS Illumina MiSeq platform. Furthermore, the full-genomic sequences of co-circulating strains PCB-118, SKT-98, BD-20, NP-M51, SKT-138, and SSKT-133, which were detected in Thailand in the same year, were determined as well, as references. The whole genomes of these nine Thai human strains were amplified using a sequence-independent primer set and then sequenced successfully. Illumina MiSeq sequencing yielded 1.5 x 10^6^ reads (~120 bp average length), 1.6 x 10^6^ reads (~120 bp average length), 1.7 x 10^6^ reads (~120 bp average length), 2.1 x 10^6^ reads (~122 bp average length), 1.7 x 10^6^ reads (~119 bp average length), 1.3 x 10^6^ reads (~119 bp average length), 1.2 x 10^6^ reads (~120 bp average length), 1.7 x 10^6^ reads (~119 bp average length), and 1.5 x 10^6^ reads (~120 bp average length) for strains SKT-281, SKT-289, LS-04, PCB-118, SKT-98, BD-20, NP-M51, SKT-138, and SSKT-133, respectively. Complete or nearly complete nucleotide sequences of all the 11 segments of the nine strains could be determined. The lengths of nucleotide and deduced amino acid amino acids sequences of the 11 gene segments of strains SKT-281, SKT-289, LS-04, PCB-118, SKT-98, BD-20, NP-M51, SKT-138, and SSKT-133, with related sequence read data is summarized in S2 Table.

**Fig 2:**
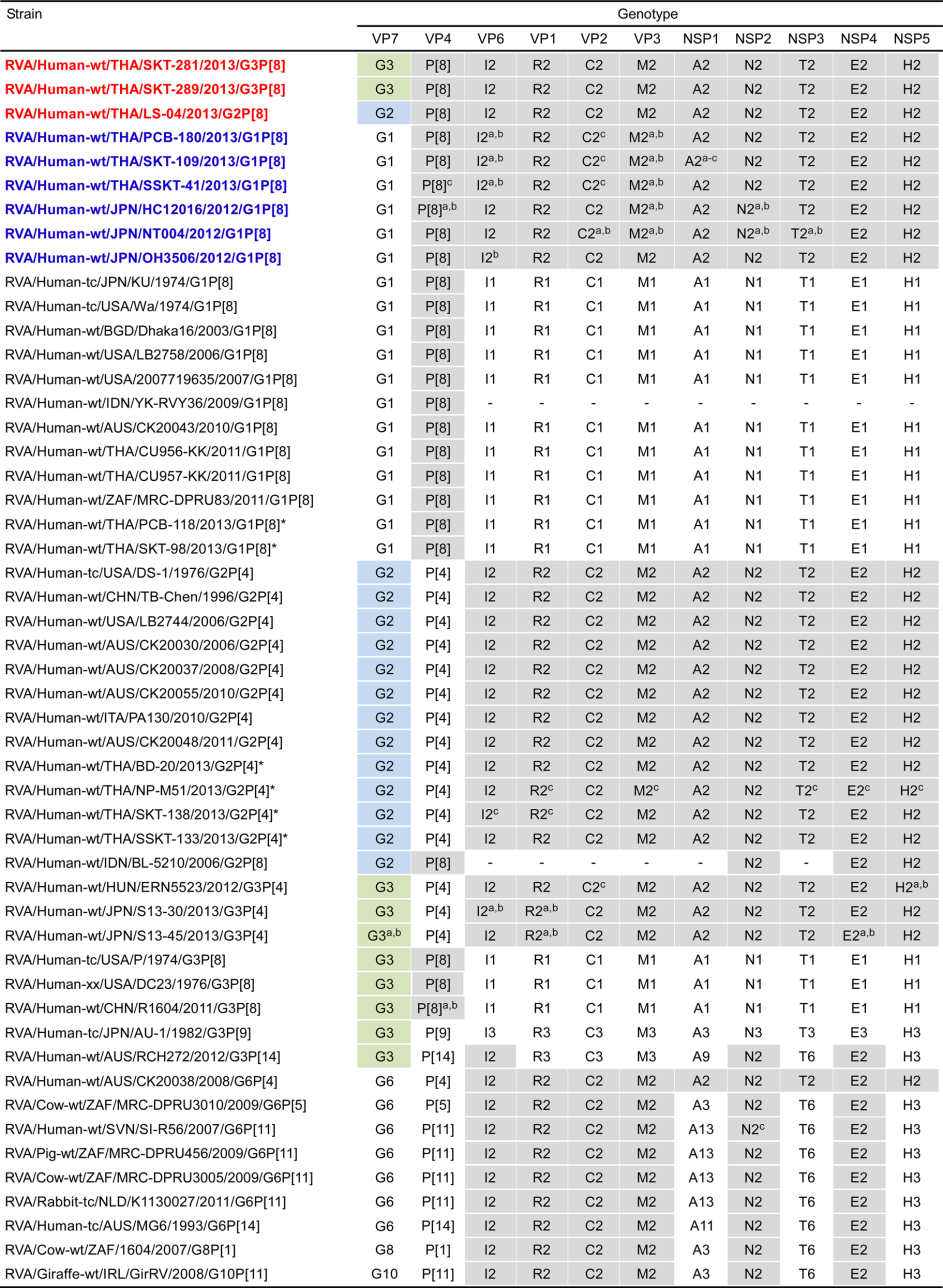
Genotype natures of the 11 gene segments of three Thai DS-1-like intergenogroup reassortant strains, SKT-281, SKT-289, and LS-04, compared with those of selected human and animal strains. Strains SKT-281, SKT-289, and LS-04 are shown in red, while DS-1-like G1P[8] strains are shown in blue. Asterisks indicate co-circulating strains PCB-118, SKT-98, BD-20, NP-M51, SKT-138, and SSKT-133. Gray shading indicates the 10 gene segments (VP4, VP6, VP1-3, and NSP1-5) with genotypes identical to those of strains SKT-281, SKT-289, and LS-04. Green shading indicates the VP7 gene segments with a G3 genotype identical to those of strains SKT-281 and SKT-289. Blue shading indicates the VP7 gene segments with a G2 genotype identical to that of strain LS-04. “−” indicates that no sequence data were available in the DDBJ and EMBL/GenBank data libraries. ^a^The gene segments that are most similar to those of strain SKT-281. ^b^The gene segments that are most similar to those of strain SKT-289. ^c^The gene segments that are most similar to those of strain LS-04.

The 11 genes of strains SKT-281, SKT-289, and LS-04 were assigned as G3-P[8]-I2-R2-C2-M2-A2-N2-T2-E2-H2, G3-P[8]-I2-R2-C2-M2-A2-N2-T2-E2-H2, and G2-P[8]-I2-R2-C2-M2-A2-N2-T2-E2-H2, respectively (Fig 2). The three Thai strains were confirmed to have G3P[8] (strains SKT-281 and SKT-289) and G2P[8] genotypes (strain LS-04), and a DS-1-like genetic backbone, as indicated on PCR-based G and P genotyping, and electropherotyping, respectively (Tacharoenmuang et al., in preparation). Thus, strains SKT-281, SKT-289, and LS-04 were named RVA/Human-wt/THA/SKT-281/2013/G3P[8], RVA/Human-wt/THA/SKT-289/2013/G3P[8], and RVA/Human-wt/THA/LS-04/2013/G2P[8], respectively, according to the guidelines for the uniformity of RVAs proposed by the RCWG. Comparison of the complete genotype constellations of strains SKT-281, SKT-289, and LS-04 with those of other G2, G3, and non-G2/G3 strains is shown in Fig 2. With the exception of the G genotype, strains SKT-281, SKT-289, and LS-04 had a unique genotype constellation, (P[8]-I2-R2-C2-M2-A2-N2-T2-E2-H2), which is commonly found in DS-1-like G1P[8] strains [13, 15, 16]. Thus, the complete genotype constellations of strains SKT-281, SKT-289, and LS-04 are mostly identical to those of DS-1-like G1P[8] strains. Furthermore, as suggested by the genomic dsRNA profiles observed on PAGE analysis (Fig 1), strains SKT-281 and SKT-289 exhibited extremely high nucleotide sequence identities (99.9–100%) to each other for all the 11 gene segments (S1 Table). However, the nucleotide sequence identities of the VP7 and other 10 gene segments (VP4, VP6, VP1-3, and NSP1-5) of strain LS-04 to those of strains SKT-281 and SKT-289 were comparatively low (73.4 and 86.6–99.5%, respectively) (S1 Table). In contrast, the 11 genes of the locally circulating strains were assigned as G1-P[8]-I1-R1-C1-M1-A1-N1-T1-E1-H1 (strains PCB-118 and SKT-98) and G2-P[4]-I2-R2-C2-M2-A2-N2-T2-E2-H2 (strains BD-20, NP-M51, SKT-138, and SSKT-133) (Fig 2), and thus named RVA/Human-wt/THA/PCB-118/2013/G1P[8], RVA/Human-wt/THA/SKT-98/2013/G1P[8], RVA/Human-wt/THA/BD-20/2013/G2P[4], RVA/Human-wt/THA/NP-M51/2013/G2P[4], RVA/Human-wt/THA/SKT-138/2013/G2P[4], and RVA/Human-wt/THA/SSKT-133/2013/G2P[4], respectively. None of these six locally circulating strains was shown to be an intergenogroup reassortant, but instead to have authentic genogroup 1 or 2 genes. Therefore, the complete genotype constellations of the six co-circulating strains are identical to those of Wa-like G1P[8] or DS-1-like G2P[4] rotaviruses.

### Phylogenetic analyses

We next constructed phylogenetic trees using the full-genome sequence for each of the 11 gene segments because phylogenetic analysis of RVA nucleotide sequences provides direct evidence of their relatedness to those of other strains, even within the same genotype [3, 5, 10] (Figs 3–16). In this study, we employed strains HC12016 [15], NT004 [13], and OH3506 [14] as representative Japanese DS-1-like G1P[8] strains, which were analyzed in three independent studies in 2014.

**Fig 3:**
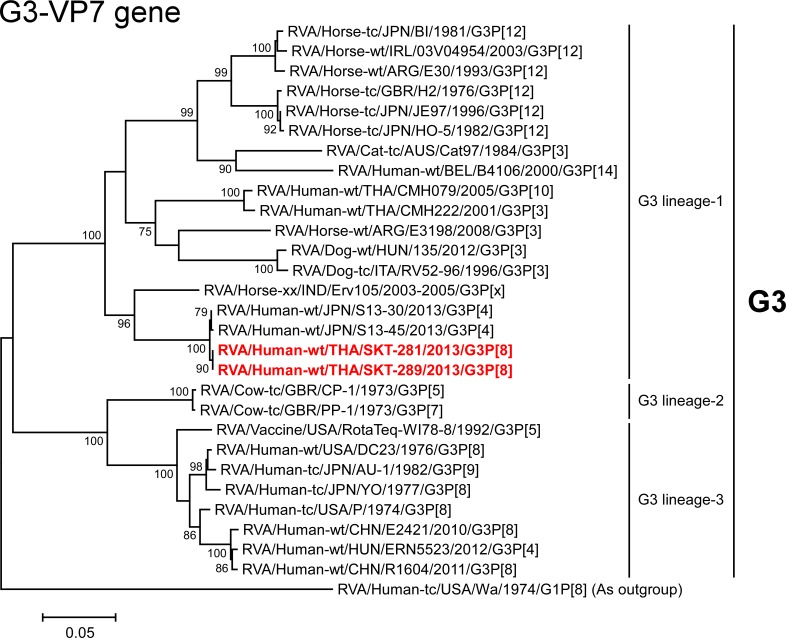
Phylogenetic tree constructed from the nucleotide sequences of the G3-VP7 genes of strains SKT-281 and SKT-289, and representative RVA strains. In the tree, the positions of strains SKT-281 and SKT-289 are shown in red. Bootstrap values of <75% are not shown. Scale bars: 0.05 substitutions per nucleotide.

**Fig 4:**
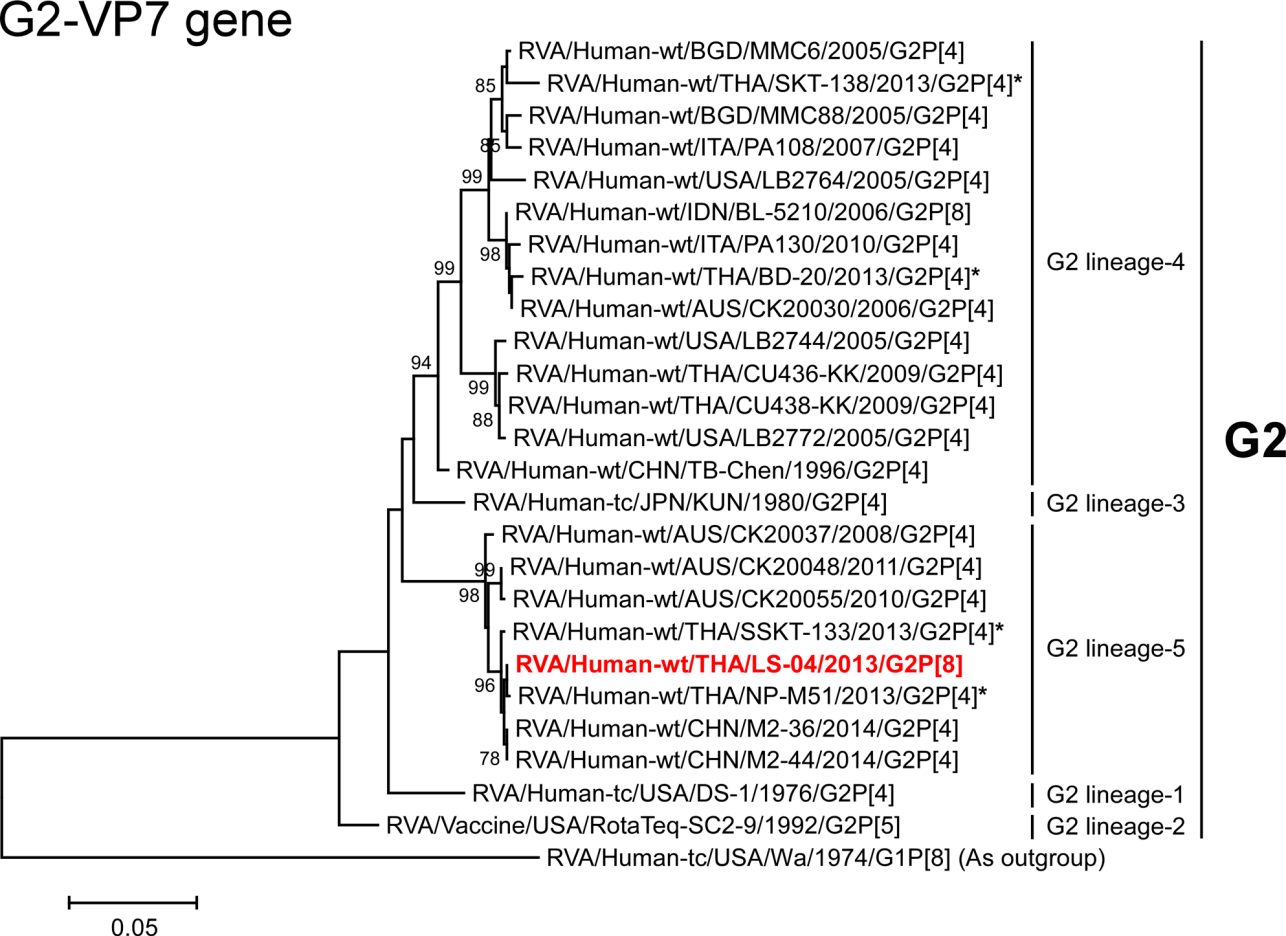
Phylogenetic tree constructed from the nucleotide sequences of the G2-VP7 genes of strain LS-04 and representative RVA strains. In the tree, the position of strain LS-04 is shown in red. Asterisks indicate co-circulating strains BD-20, NP-M51, SKT-138, and SSKT-133. Bootstrap values of <75% are not shown. Scale bars: 0.05 substitutions per nucleotide.

**Fig 5:**
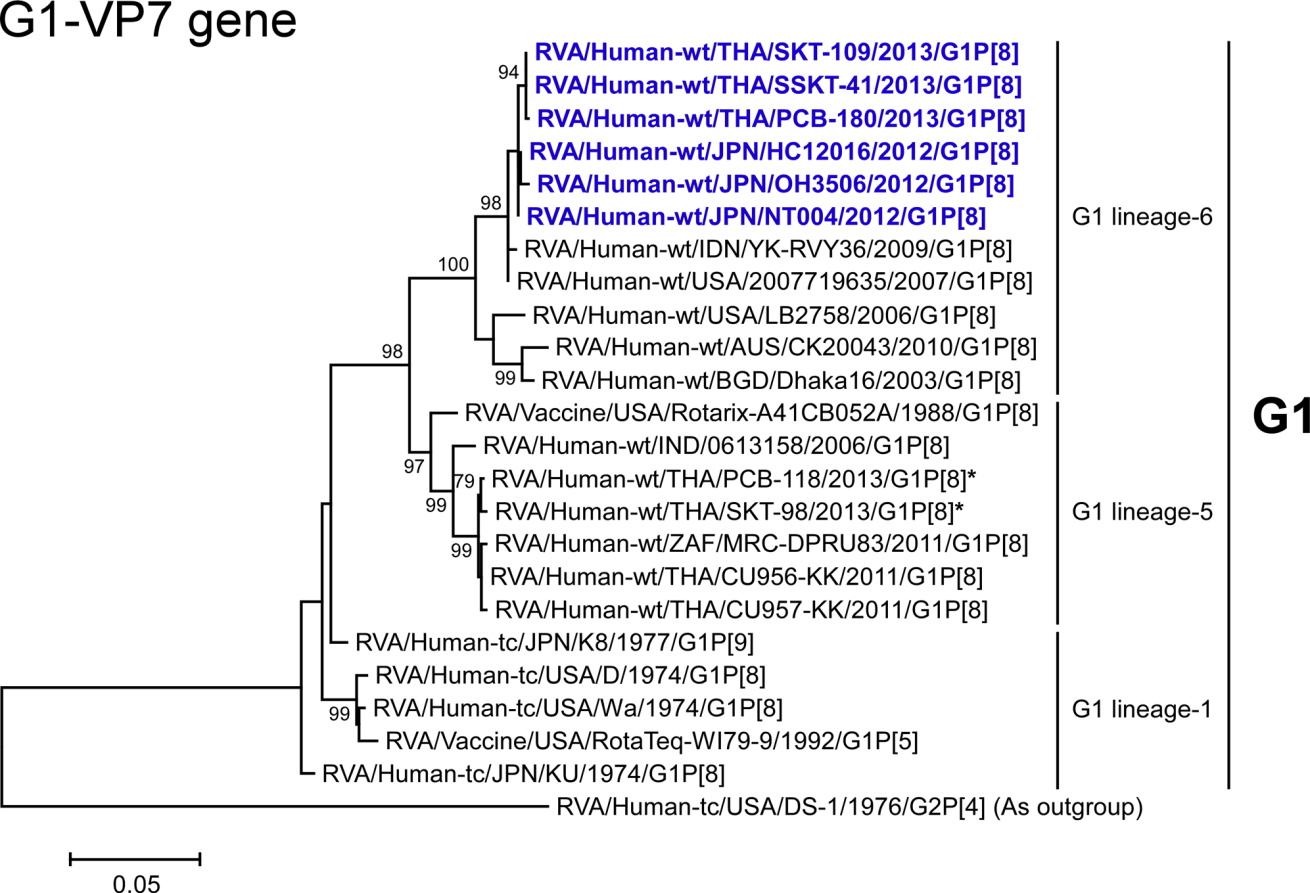
Phylogenetic tree constructed from the nucleotide sequences of the G1-VP7 genes of co-circulating strains PCB-118 and SKT-98, and representative RVA strains. In the tree, the positions of DS-1-like G1P[8] strains are shown in blue. Asterisks indicate co-circulating strains PCB-118 and SKT-98. Bootstrap values of <75% are not shown. Scale bars: 0.05 substitutions per nucleotide.

**Fig 6:**
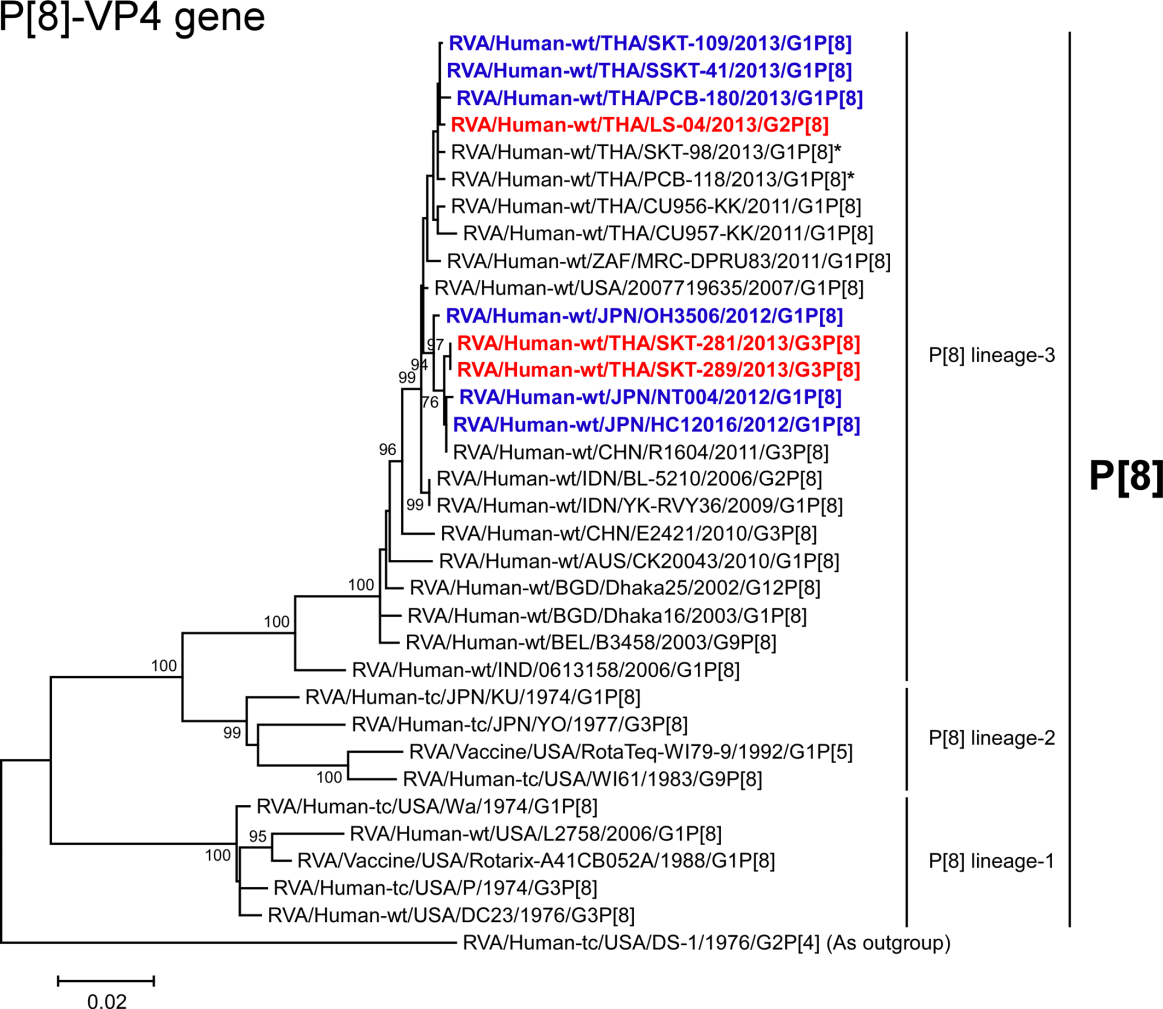
Phylogenetic tree constructed from the nucleotide sequences of the P[8]-VP4 genes of strains SKT-281, SKT-289, and LS-04, and representative RVA strains. In the tree, the positions of strains SKT-281, SKT-289, and LS-04 are shown in red, while those of DS-1-like G1P[8] strains are shown in blue. Asterisks indicate co-circulating strains PCB-118 and SKT-98. Bootstrap values of <75% are not shown. Scale bars: 0.02 substitutions per nucleotide.

**Fig 7:**
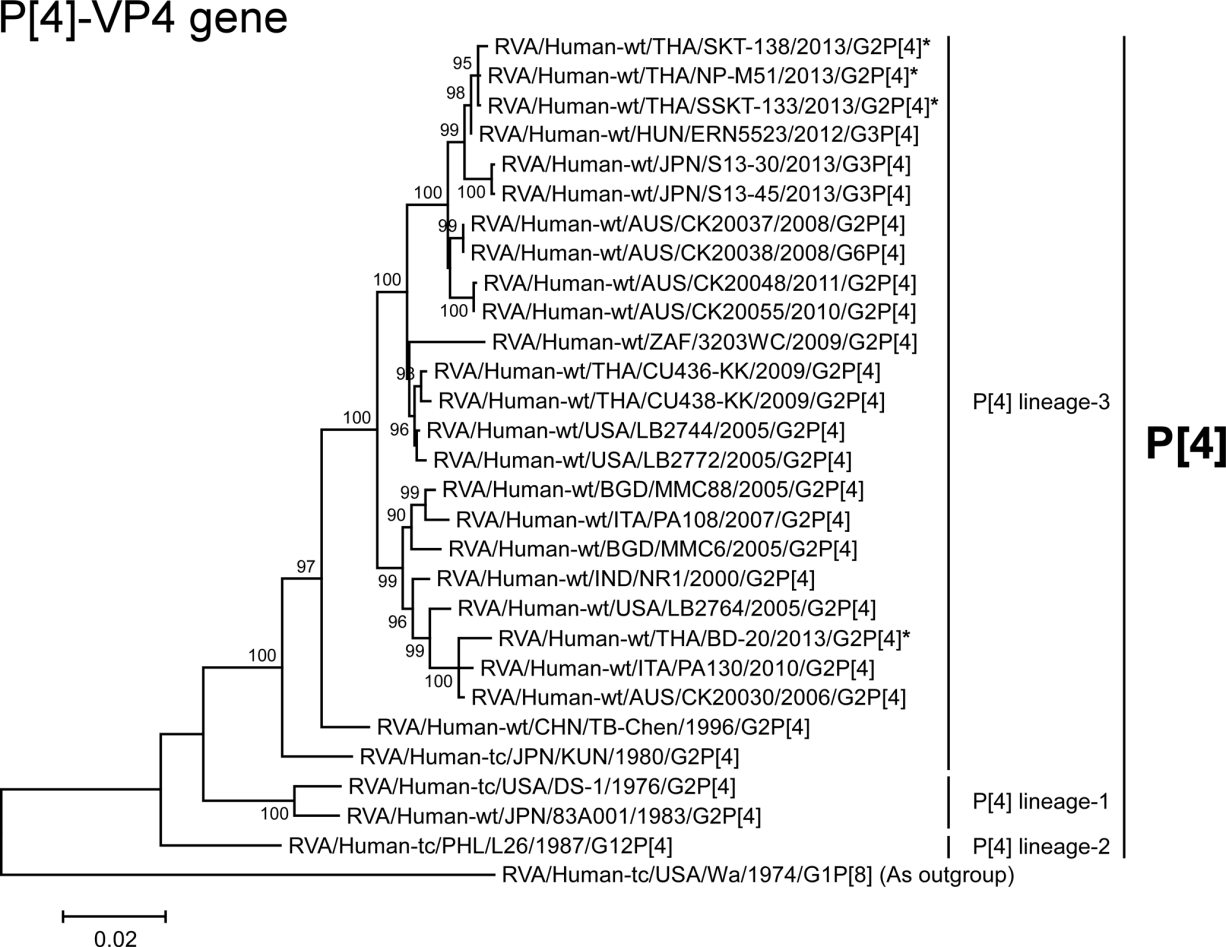
Phylogenetic tree constructed from the nucleotide sequences of the P[4]-VP4 genes of co-circulating strains BD-20, NP-M51, SKT-138, and SSKT-133, and representative RVA strains. In the tree, asterisks indicate co-circulating strains BD-20, NP-M51, SKT-138, and SSKT-133. Bootstrap values of <75% are not shown. Scale bars: 0.02 substitutions per nucleotide.

**Fig 8:**
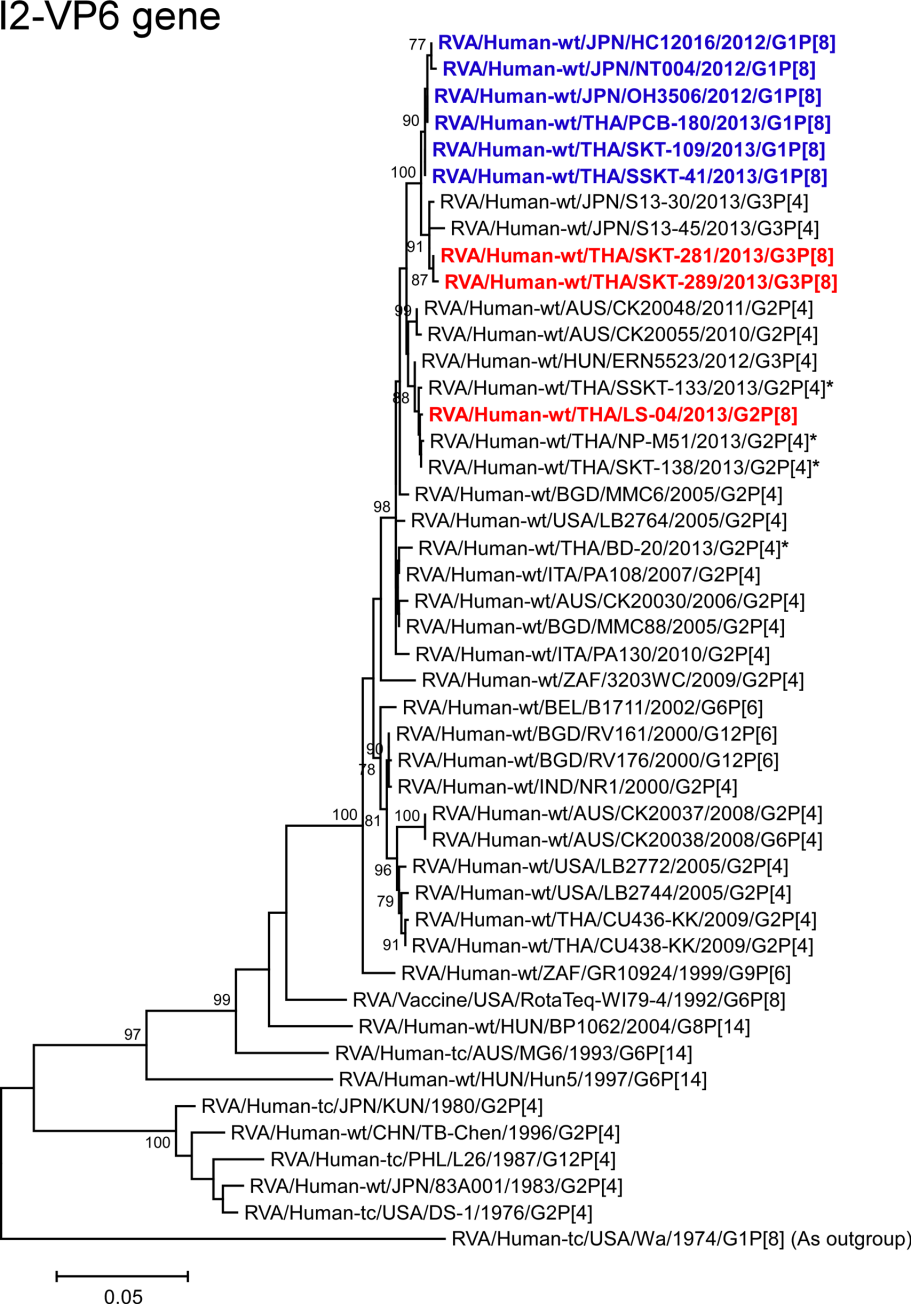
Phylogenetic tree constructed from the nucleotide sequences of the I2-VP6 genes of strains SKT-281, SKT-289, and LS-04, and representative RVA strains. In the tree, the positions of strains SKT-281, SKT-289, and LS-04 are shown in red, while those of DS-1-like G1P[8] strains are shown in blue. Asterisks indicate co-circulating strains BD-20, NP-M51, SKT-138, and SSKT-133. Bootstrap values of <75% are not shown. Scale bars: 0.05 substitutions per nucleotide.

**Fig 9:**
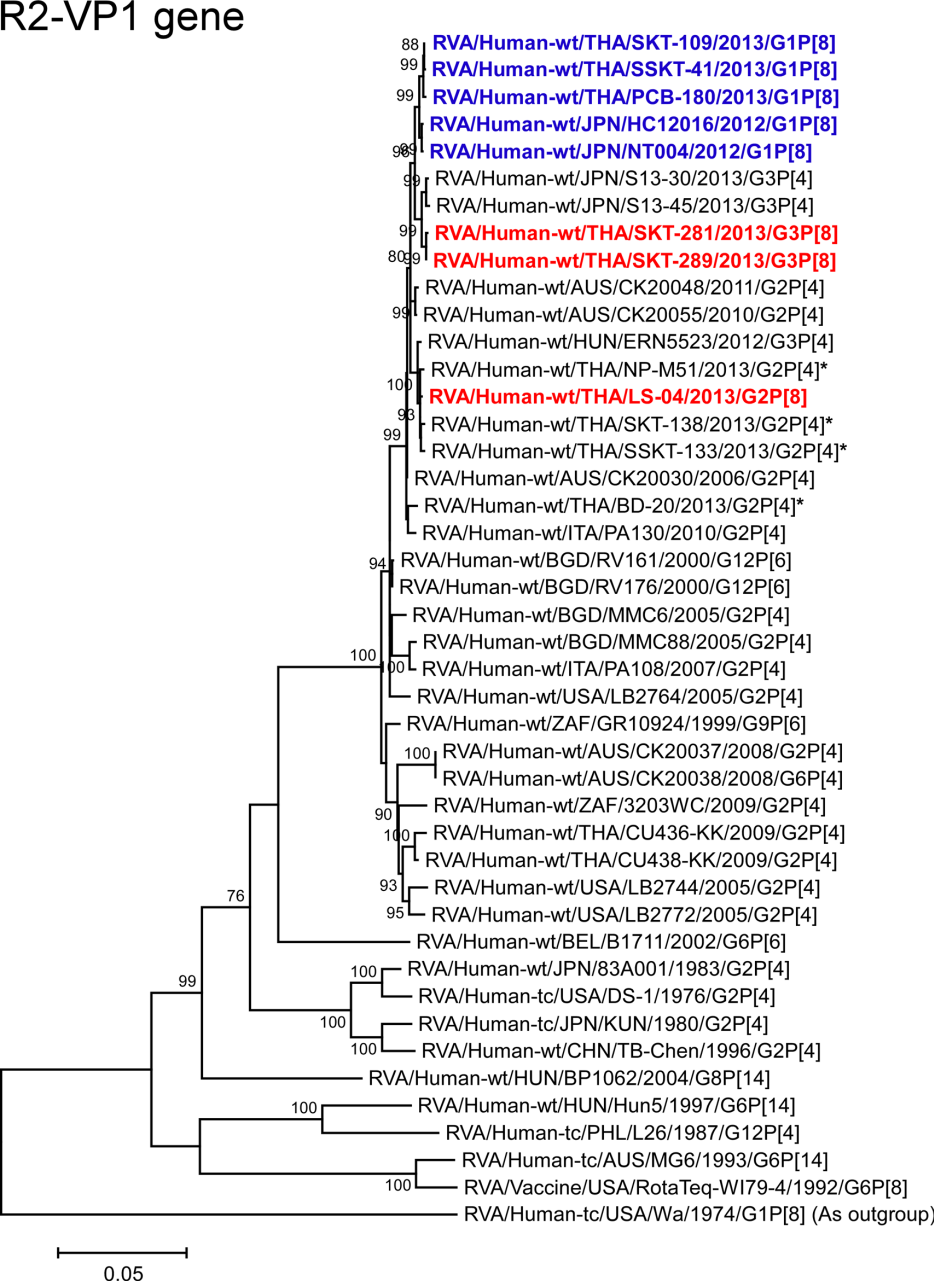
Phylogenetic tree constructed from the nucleotide sequences of the R2-VP1 genes of strains SKT-281, SKT-289, and LS-04, and representative RVA strains. See legend of Fig 8. Scale bars: 0.05 substitutions per nucleotide.

**Fig 10:**
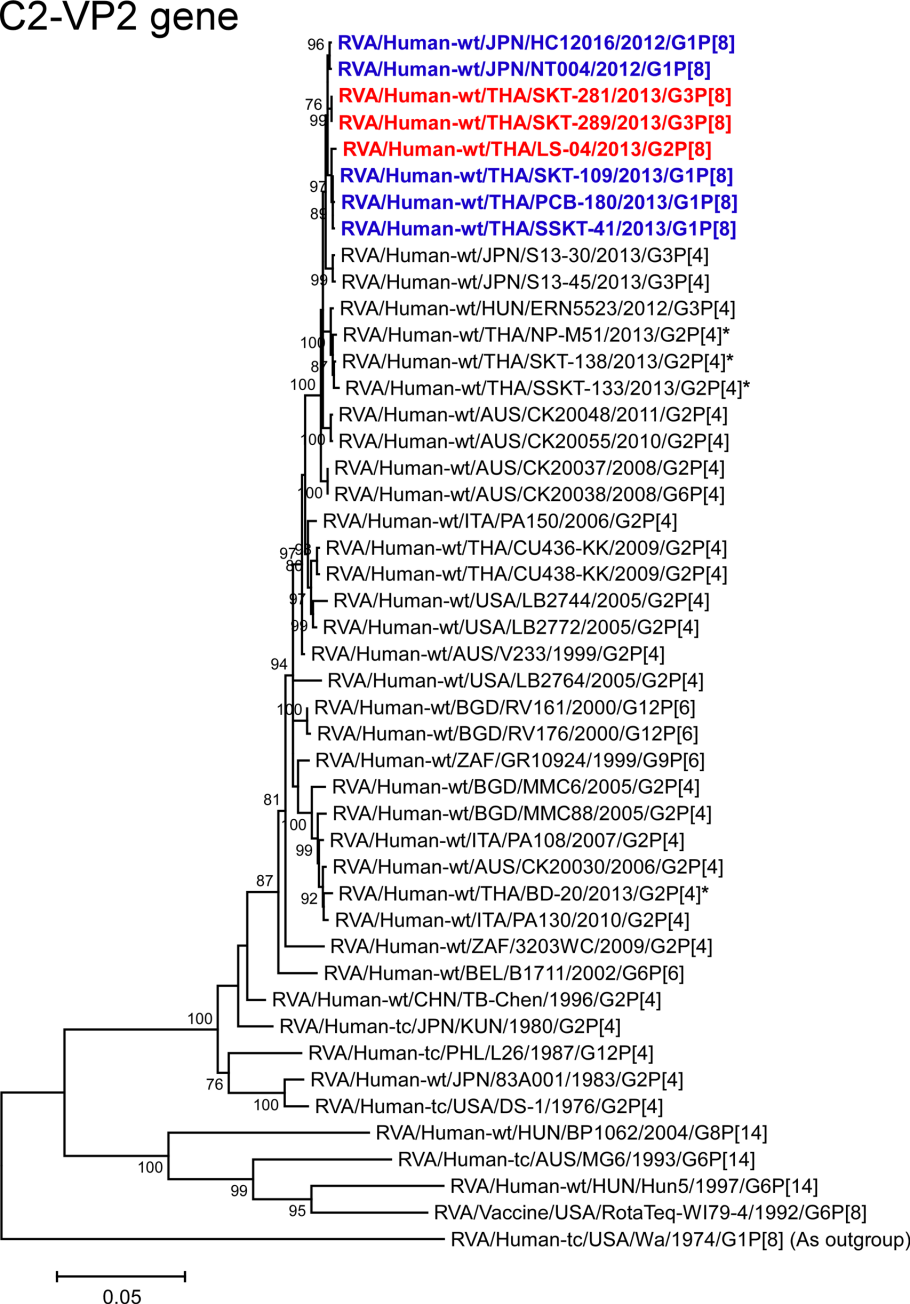
Phylogenetic tree constructed from the nucleotide sequences of the C2-VP2 genes of strains SKT-281, SKT-289, and LS-04, and representative RVA strains. See legend of Fig 8. Scale bars: 0.05 substitutions per nucleotide.

**Fig 11:**
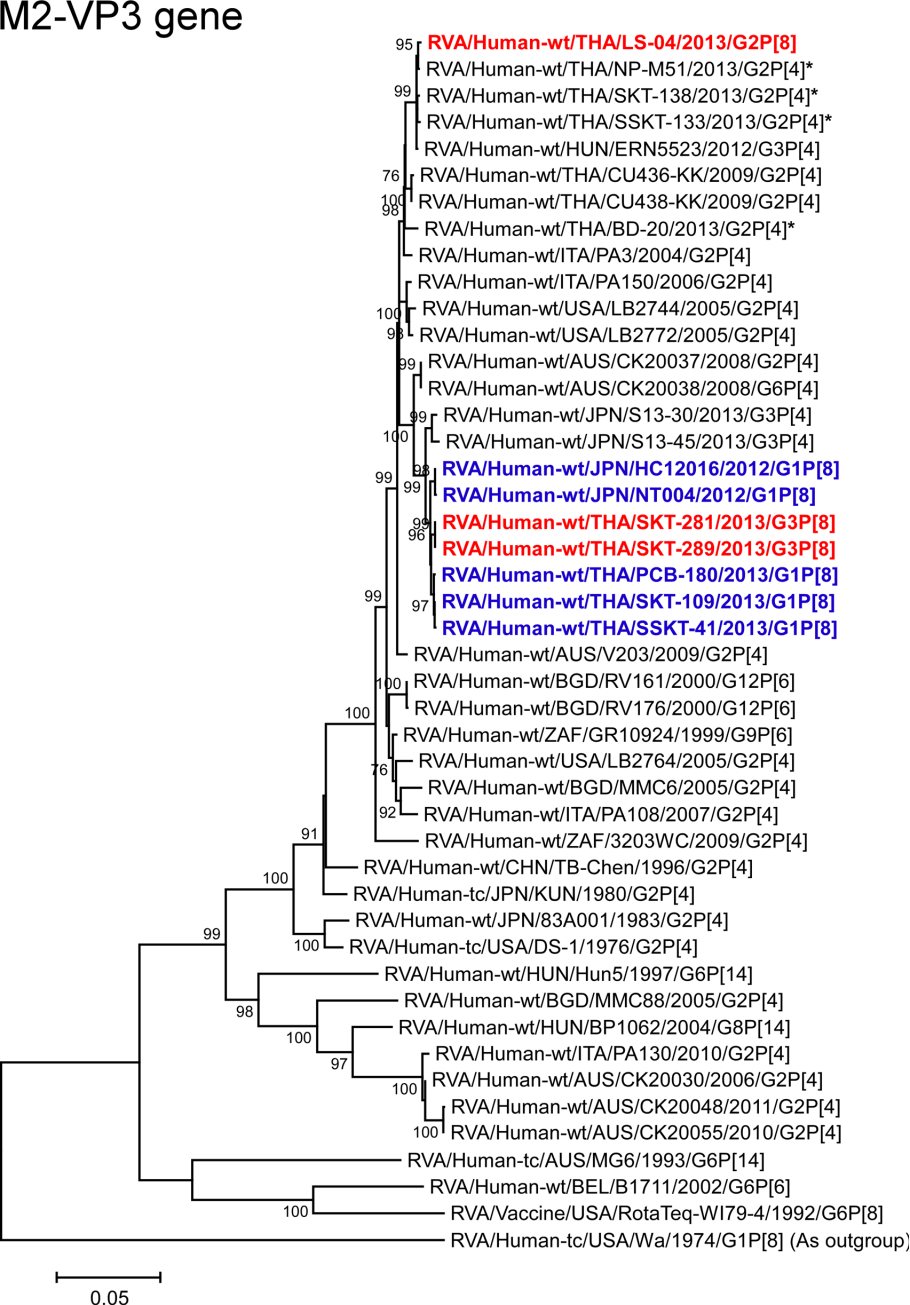
Phylogenetic tree constructed from the nucleotide sequences of the M2-VP3 genes of strains SKT-281, SKT-289, and LS-04, and representative RVA strains. See legend of Fig 8. Scale bars: 0.05 substitutions per nucleotide.

**Fig 12:**
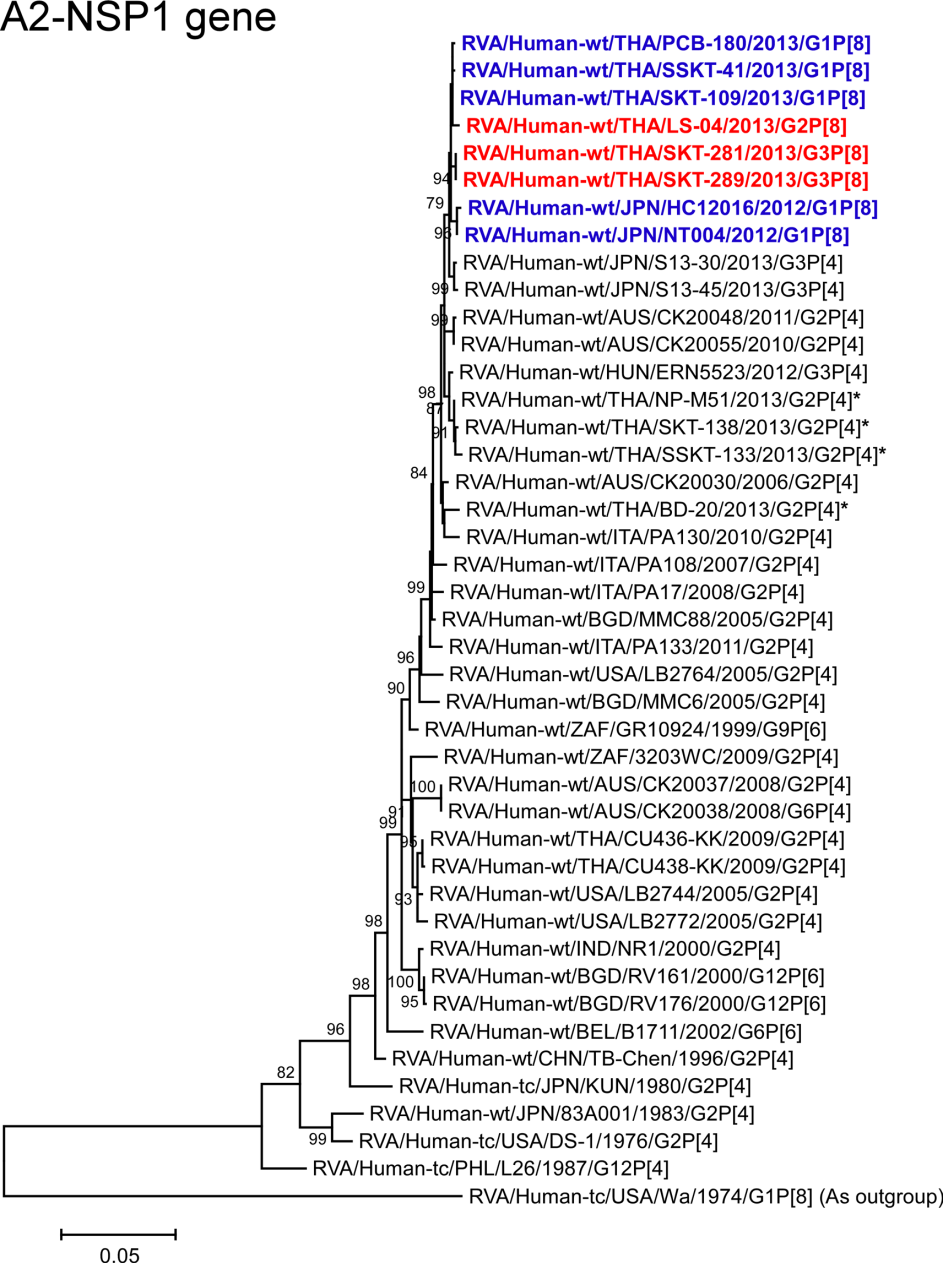
Phylogenetic tree constructed from the nucleotide sequences of the A2-NSP1 genes of strains SKT-281, SKT-289, and LS-04, and representative RVA strains. See legend of Fig 8. Scale bars: 0.05 substitutions per nucleotide.

**Fig 13:**
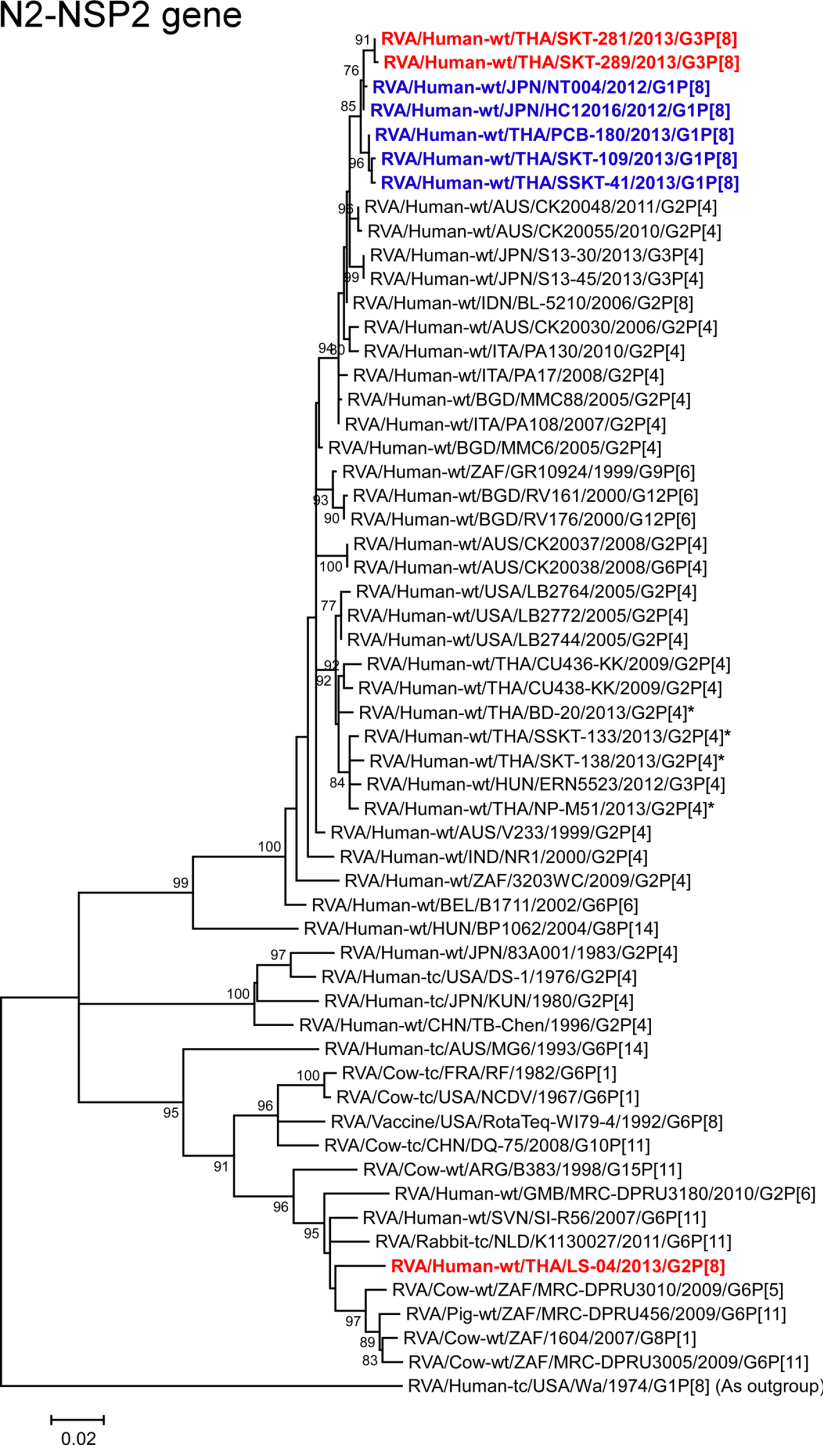
Phylogenetic tree constructed from the nucleotide sequences of the N2-NSP2 genes of strains SKT-281, SKT-289, and LS-04, and representative RVA strains. See legend of Fig 8. Scale bars: 0.02 substitutions per nucleotide.

**Fig 14:**
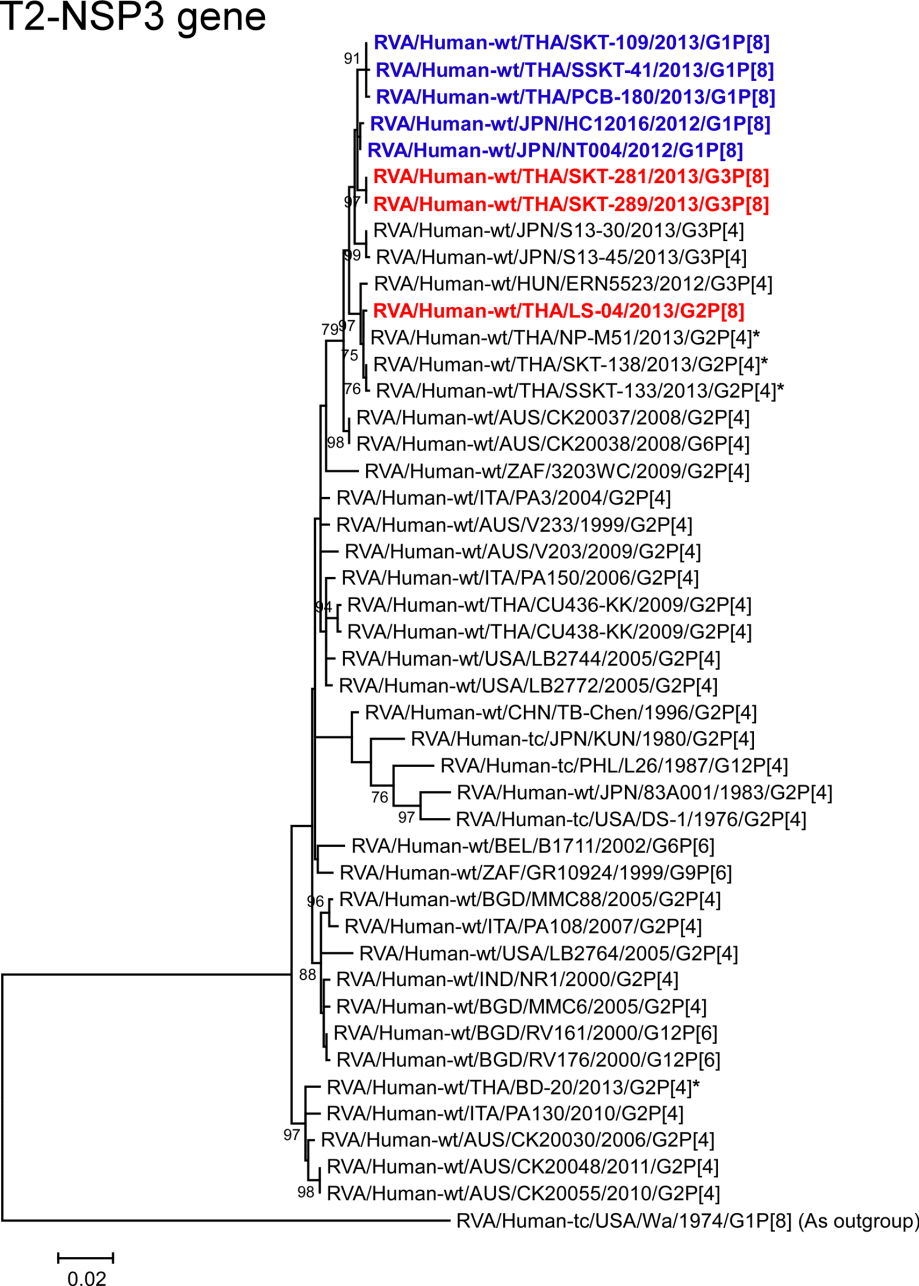
Phylogenetic tree constructed from the nucleotide sequences of the T2-NSP3 genes of strains SKT-281, SKT-289, and LS-04, and representative RVA strains. See legend of Fig 8. Scale bars: 0.02 substitutions per nucleotide.

**Fig 15:**
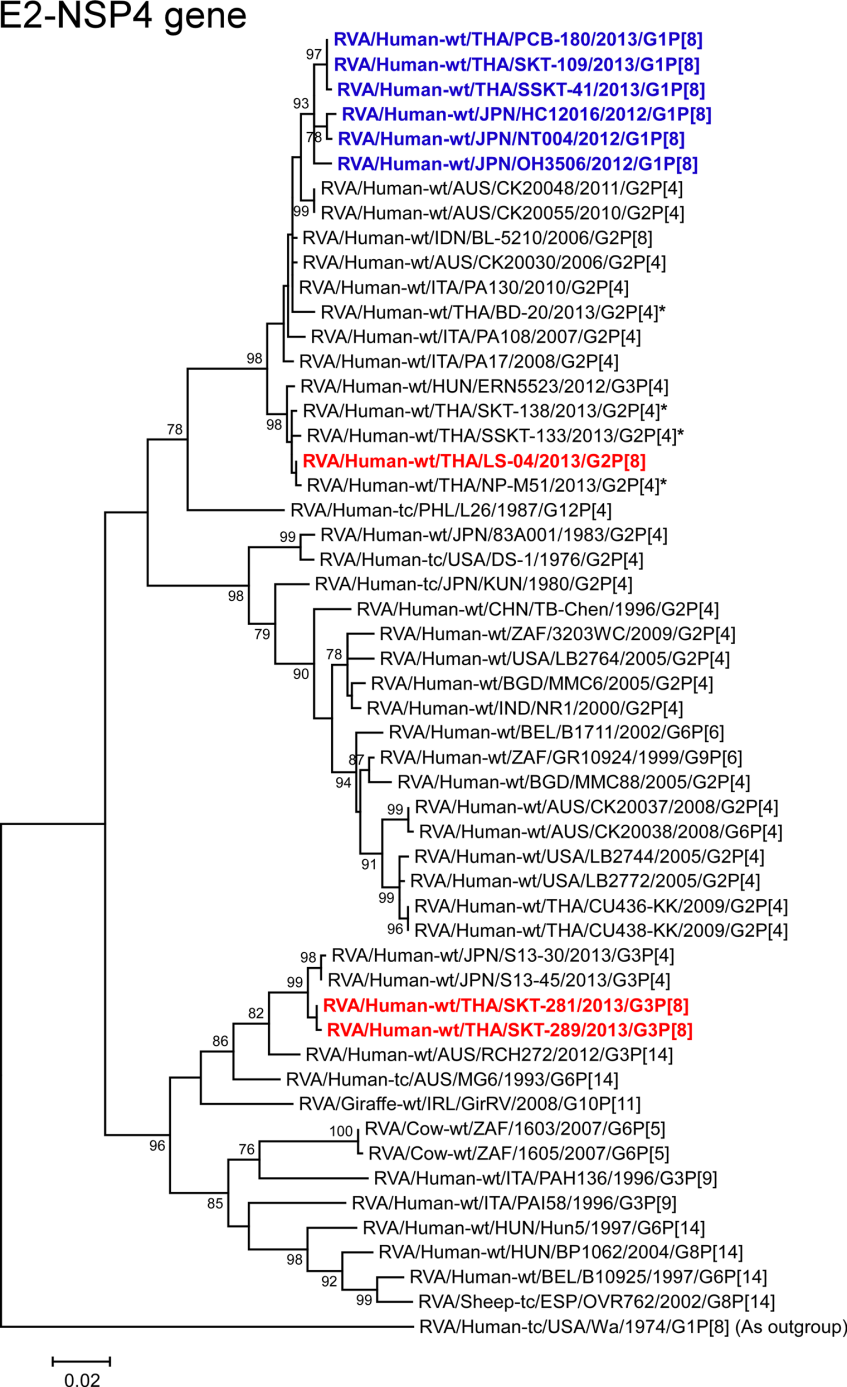
Phylogenetic tree constructed from the nucleotide sequences of the E2-NSP4 genes of strains SKT-281, SKT-289, and LS-04, and representative RVA strains. See legend of Fig 8. Scale bars: 0.02 substitutions per nucleotide.

**Fig 16:**
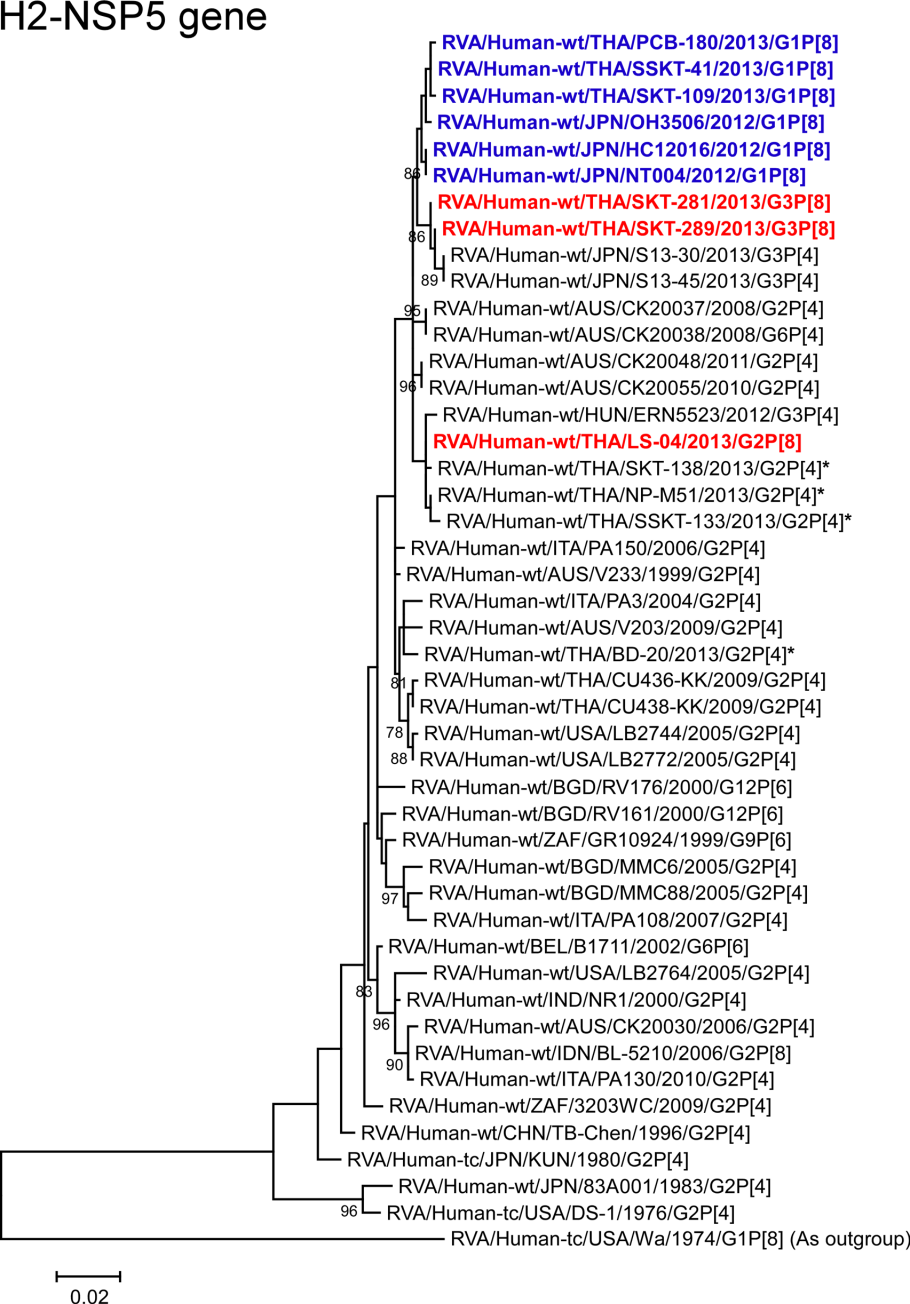
Phylogenetic tree constructed from the nucleotide sequences of the H2-NSP5 genes of strains SKT-281, SKT-289, and LS-04, and representative RVA strains. See legend of Fig 8. Scale bars: 0.02 substitutions per nucleotide.

The VP7 genes of strains SKT-281 and SKT-289 exhibited the highest nucleotide sequence identities (99.1%) with that of Japanese equine-like human strain S13-45 (G3P[4]) [25], and comparable identities (99.0%) with Japanese equine-like human strain S13-30 (G3P[4]) [25]. On phylogenetic analysis, strains SKT-281 and SKT-289 were shown to be closely related with strains S13-30 and S13-45 in a common branch with Indian equine strain Erv105 (G3P[x]) [26] within animal-like G3 lineage-1 (Fig 3). On the other hand, the VP7 gene of strain LS-04 exhibited the maximum nucleotide sequence identity (99.8%) with co-circulating human strain SSKT-133 (G2P[4]), and comparable identity (99.7%) with co-circulating human strain NP-M51 (G2P[4]). Phylogenetically, strain LS-04 was shown to be closely related with these Thai human G2P[4] strains and Chinese human strains M2-36 (G2P[4]) and M2-44 (G2P[4]) in G2 lineage-5 (Fig 4).

The VP4 genes of strains SKT-281 and SKT-289 exhibited the maximum nucleotide sequence identities (99.8%) with the cognate genes of Japanese DS-1-like G1P[8] strain HC12016 and Chinese human strain R1604 (G3P[8]) [27], and comparable identities (99.7%) with Japanese DS-1-like G1P[8] strains NT004 and OH3506. Phylogenetically, strains SKT-281 and SKT-289 were found to form a cluster with the above-mentioned P[8] strains from Asia (Fig 6). In contrast, the VP4 gene of strain LS-04 showed the highest nucleotide sequence identity (99.9%) with Thai DS-1-like G1P[8] strain SSKT-41, and comparable identity (99.7–99.8%) with Thai DS-1-like G1P[8] strains (PCB-180 and SKT-109), and co-circulating human strains PCB-118 (G1P[8]) and SKT-98 (G1P[8]). On phylogenetic analysis, strain LS-04 was found to form a cluster with these Thai P[8] strains, slightly away from the cluster comprising strains SKT-281 and SKT-289, and Japanese DS-1-like G1P[8] strains in P[8] lineage-3 (Fig 6).

The VP6 genes of strains SKT-281 and SKT-289 showed the maximum nucleotide identities (99.4 and 99.3%, respectively) with the VP6 genes of Thai DS-1-like G1P[8] strains (PCB-180, SKT-109, and SSKT-41), Japanese DS-1-like G1P[8] strain OH3506, and Japanese equine-like human strain S13-30 (G3P[4]), and comparable identities (99.0–99.2%) with Japanese DS-1-like G1P[8] strains HC12016 and NT004. Phylogenetically, however, strains SKT-281 and SKT-289 were found to be closely related with strain S13-30 and Japanese equine-like human strain S13-45 in a common branch with these DS-1-like G1P[8] strains from Thailand and Japan (Fig 8). On the other hand, the VP6 gene of strain LS-04 exhibited complete nucleotide sequence identity (100%) with co-circulating human strain SKT-138 (G2P[4]), and comparable identity (99.8–99.9%) with co-circulating human strains (NP-M51 (G2P[4]) and SSKT-133 (G2P[4])) and Hungarian human strain ERN5523 (G3P[4]) [28]. On phylogenetic analysis, strain LS-04 was shown to form a cluster with these strains (Fig 8).

The VP1 genes of strains SKT-281 and SKT-289 exhibited the highest nucleotide sequence identities (99.5%) with the cognate genes of Japanese equine-like human strains S13-30 (G3P[4]) and S13-45 (G3P[4]), and comparable identities (99.2–99.3%) with Japanese DS-1-like G1P[8] strains (HC12016 and NT004), Thai DS-1-like G1P[8] strains (PCB-180, SKT-109, and SSKT-41), and Australian human strain CK20055 (G2P[4]). Phylogenetically, SKT-281 and SKT-289 were found to be closely related with strains S13-30 and S13-45 near these DS-1-like G1P[8] strains from Japan and Thailand (Fig 9). On the other hand, the VP1 gene of strain LS-04 showed the maximum nucleotide sequence identity (99.8%) with co-circulating human strains NP-M51 (G2P[4]) and SKT-138 (G2P[4]), and comparable identity (99.7%) with co-circulating human strain SSKT-133 (G2P[4]) and Hungarian human strain ERN5523 (G3P[4]). On phylogenetic analysis, strain LS-04 formed a cluster with these strains (Fig 9).

The VP2 genes of strains SKT-281 and SKT-289 exhibited the maximum nucleotide sequence identities (99.7%) with the VP2 gene of Japanese DS-1-like G1P[8] strain NT004, and comparable identities (99.5–99.6%) with strain LS-04, Thai DS-1-like G1P[8] strains (PCB-180, SKT-109, and SSKT-41), and Japanese DS-1-like G1P[8] strain HC12016. Conversely, the VP2 gene of strain LS-04 showed the highest nucleotide sequence identity (99.8%) with Thai DS-1-like G1P[8] strains PCB-180, SKT-109, and SSKT-41, and comparable identity (99.5–99.6%) with strains SKT-281 and SKT-289, and Japanese DS-1-like G1P[8] strains HC12016 and NT004. On phylogenetic analysis, strains SKT-281, SKT-289, and LS-04 were found to be closely related with these DS-1-like G1P[8] strains from Japan and Thailand (Fig 10).

The VP3 genes of strains SKT-281 and SKT-289 exhibited the maximum nucleotide sequence identities (99.6%) with the VP3 genes of Thai DS-1-like G1P[8] strains (PCB-180, SKT-109, and SSKT-41), and Japanese DS-1-like G1P[8] strains HC12016 and NT004. On phylogenetic analysis, strains SKT-281 and SKT-289 were found to form a cluster with these DS-1-like G1P[8] strains from Thailand and Japan (Fig 11). In contrast, the VP3 gene of strain LS-04 showed the maximum nucleotide sequence identity (99.8%) with co-circulating human strain NP-M51 (G2P[4]) and Hungarian human strain ERN5523 (G3P[4]), and comparable identity (99.6%) with co-circulating human strains SKT-138 (G2P[4]) and SSKT-133 (G2P[4]). On phylogenetic analysis, strain LS-04 was shown to form a cluster with these strains (Fig 11).

All the NSP1 genes of strains SKT-281, SKT-289, and LS-04 showed the maximum nucleotide sequence identities (99.7%, 99.7%, and 99.8%, respectively) with that of Thai DS-1-like G1P[8] strain SKT-109, and comparable identities (99.5–99.6%, 99.4–99.6%, and 99.4–99.7%, respectively) with Thai DS-1-like G1P[8] strains (PCB-180 and SSKT-41), and Japanese DS-1-like G1P[8] strains HC12016 and NT004. On phylogenetic analysis, strains SKT-281, SKT-289, and LS-04 were found to be closely related with these DS-1-like G1P[8] strains from Thailand and Japan (Fig 12).

The NSP2 genes of strains SKT-281 and SKT-289 showed the highest nucleotide sequence identities (99.5%) with the cognate genes of Japanese DS-1-like G1P[8] strains HC12016 and NT004, and somewhat lower identities (99.0–99.2%) with Thai DS-1-like G1P[8] strains (PCB-180, SKT-109, and SSKT-41) and Indonesian human strain BL-5210 (G2P[8]). Phylogenetically, strains SKT-281 and SKT-289 were found to be closely related with these DS-1-like G1P[8] strains from Japan and Thailand (Fig 13). On the other hand, the NSP2 gene of strain LS-04 exhibited the maximum nucleotide sequence identity (97.2%) with Slovenian bovine-like human strain SI-R56 (G6P[11]) [29], and somewhat lower identity (96.4–96.6%) with South African bovine strains (MRC-DPRU3010 (G6P[5]) [30], MRC-DPRU3005 (G6P[11]), and 1604 (G8P[1]) [31], Dutch bovine-like lapine strain K1130027 (G6P[11]) [32], and South African bovine-like porcine strain MRC-DPRU456 (G6P[11]). On phylogenetic analysis, strain LS-04 was found to be clustered with these bovine, bovine-like human, bovine-like lapine, and bovine-like porcine strains within the bovine-like N2 subcluster (Fig 13).

The NSP3 genes of strains SKT-281 and SKT-289 exhibited the maximum nucleotide sequence identities (99.7%) with the NSP3 gene of Japanese DS-1-like G1P[8] strain NT004, and comparable identities (99.3–99.5%) with Japanese DS-1-like G1P[8] strain HC12016, and Thai DS-1-like G1P[8] strains PCB-180, SKT-109, and SSKT-41. On phylogenetic analysis, strains SKT-281 and SKT-289 were found to be closely related with these DS-1-like G1P[8] strains from Japan and Thailand (Fig 14). In contrast, the NSP3 gene of strain LS-04 showed the highest nucleotide sequence identity (99.9%) with co-circulating human strain NP-M51 (G2P[4]), and comparable identity (99.6–99.7%) with co-circulating human strains (SKT-138 and SSKT-133) and Hungarian human strain ERN5523 (G3P[4]). On phylogenetic analysis, strain LS-04 was shown to form a cluster with these strains (Fig 14).

The NSP4 genes of strains SKT-281 and SKT-289 exhibited the highest nucleotide sequence identities (99.3%) with the cognate gene of Japanese equine-like human strain S13-45 (G3P[4]), and comparable identities (99.1%) with Japanese equine-like human strain S13-30 (G3P[4]). Phylogenetically, strains SKT-281 and SKT-289 were found to be closely related with strains S13-30 and S13-45 in a common branch with Australian bovine-like human strains (RCH272 (G3P[14]) [33] and MG6 (G6P[14]) [34]) and Irish bovine-like giraffe strain GirRV (G10P[11]) [35] within the bovine-like E2 subcluster (Fig 15). In contrast, the NSP4 gene of strain LS-04 showed complete nucleotide sequence identity (100%) with co-circulating human strain NP-M51 (G2P[4]), and comparable identity (99.5–99.7%) with co-circulating human strains (SKT-138 (G2P[4]) and SSKT-133 (G2P[4])) and Hungarian human strain ERN5523 (G3P[4]). On phylogenetic analysis, strain LS-04 was shown to form a cluster with these strains (Fig 15).

The NSP5 genes of strains SKT-281 and SKT-289 showed the maximum nucleotide sequence identities (99.5%) with that of Hungarian human strain ERN5523 (G3P[4]), and comparable identities (99.1–99.3%) with Thai DS-1-like G1P[8] strains (PCB-180, SKT-109, and SSKT-41), Japanese DS-1-like G1P[8] strains (HC12016, NT004 and OH3506), Japanese equine-like human strains (S13-30 (G3P[4]) and S13-45 (G3P[4])), and Australian human strains CK20048 (G2P[4]) and CK20055 (G2P[4]). On phylogenetic analysis, however, strains SKT-281 and SKT-289 were found to be closely related with strains S13-30 and S13-45 in a common branch with these DS-1-like G1P[8] strains from Thailand and Japan (Fig 16). On the other hand, the NSP5 gene of strain LS-04 exhibited complete nucleotide sequence identity (100%) with co-circulating human strain NP-M51 (G2P[4]), and comparable identity (99.6–99.8%) with co-circulating human strains (SKT-138 (G2P[4]) and SSKT-133 (G2P[4])) and Hungarian human strain ERN5523 (G3P[4]). On phylogenetic analysis, strain LS-04 was shown to form a cluster with these strains (Fig 16).

In the present study, we analyzed the whole genomes of three DS-1-like intergenogroup reassortant strains having G3P[8] (strains SKT-281 and SKT-289) and G2P[8] (strain LS-04) genotypes identified in stool samples from hospitalized children with severe gastroenteritis in Thailand. No difference in clinical presentation and severity was found among RVA genotypes (Tacharoenmuang et al., in preparation). All the three strains showed unique genotype constellations comprising mixtures of genogroup 1 and 2 genes: G3-P[8]-I2-R2-C2-M2-A2-N2-T2-E2-H2 (strains SKT-281 and SKT-289) and G2-P[8]-I2-R2-C2-M2-A2-N2-T2-E2-H2 (strain LS-04). With the exception of the G genotype, the unique genotype constellation of the three strains (P[8]-I2-R2-C2-M2-A2-N2-T2-E2-H2) is commonly shared with DS-1-like G1P[8] strains. On phylogenetic analysis, nine of the 11 genes of strains SKT-281 and SKT-289 (VP4, VP6, VP1-3, NSP1-3, and NSP5) appeared to have originated from DS-1-like G1P[8] strains, whereas the remaining VP7 and NSP4 genes were assumed to be of equine and bovine origin, respectively. Therefore, strains SKT-281 and SKT-289 seemed to have been derived through reassortment event(s) between DS-1-like G1P[8], animal-like human and/or animal rotaviruses. However, the exact origins of the VP7 and NSP4 genes of strains SKT-281 and SKT-289 could not be ascertained due to a lack of a sufficient number of representative animal-like human and animal strains as references. Furthermore, strains SKT-281 and SKT-289 were very closely related to each other in all the 11 gene segments, indicating the derivation of the two strains from a common ancestor. In contrast, seven of the 11 genes of strain LS-04 (VP7, VP6, VP1, VP3, and NSP3-5) were assumed to have originated from locally circulating DS-1-like G2P[4] human rotaviruses, while three genes (VP4, VP2, and NSP1) appeared to be derived from DS-1-like G1P[8] strains. The remaining NSP2 gene of strain LS-04 was assumed to be of bovine origin, although the exact origin of this gene of strain LS-04 could not be ascertained due to a lack of a sufficient number of representative bovine-like human and bovine strains as references. Thus, strain LS-04 appeared to be a multiple reassortment strain involving DS-1-like G1P[8], locally circulating DS-1-like G2P[4], bovine-like human, and/or bovine rotaviruses. Overall, the great genomic diversity among the DS-1-like G1P[8] strains seemed to have been generated through reassortment involving human and animal strains.

Of note is that the VP4 gene of strain LS-04 showed the closest relationship with the cognate genes of locally circulating Wa-like G1P[8] strains, as well as Thai DS-1-like G1P[8] strains, being slightly away from the cluster comprising strains SKT-281 and SKT-289, and Japanese DS-1-like G1P[8] strains (Fig 6). These results might imply the occurrence of reassortment between Japanese DS-1-like G1P[8] and the locally circulating Wa-like G1P[8] strains to form Thai DS-1-like G1P[8] strains having the LS-04-like VP4 genes. Another possibility is that the DS-1-like G1P[8] strains might have originated from not a single, but at least two distinct ancestral Wa-like G1P[8] strains that possessed slightly different VP4 genes. However, global rotavirus strain collection is required to determine the exact evolutionary patterns of DS-1-like G1P[8] strains and related reassortant ones. To our knowledge, this is the first report on whole genome-based characterization of DS-1-like intergenogroup reassortant strains having G3P[8] and G2P[8] genotypes that have emerged in Thailand. We need to continue surveying the emergence of any unusual RVA strains such as DS-1-like G1P[8] strains and related reassortant ones in order to determine the relationship with the recent introduction of rotavirus vaccines.

The emergence of DS-1-like intergenogroup reassortant strains having G3P[8] and G2P[8] genotypes in Thailand may imply the constant circulation of DS-1-like G1P[8] strains and the occurrence of reassortment involving them, at least in Asia. Because DS-1-like G1P[8] strains have successfully spread in broad locations in Asia [13–16], continued surveillance of DS-1-like G1P[8] strains and related reassortant ones is required. Although most studies on RVA genotype distributions have been based on only G/P defining genes, PCR-based genotyping for non-G/P defining gene(s) or PAGE analysis would assist the identification of unusual RVA strains such as DS-1-like G1P[8] strains and related reassortant ones. Furthermore, whole genome-based analyses are essential to understand the evolutionary dynamics of emerging DS-1-like G1P[8] strains and related reassortant ones.

## Supporting Information

S1 Table Nucleotide sequence identities (%) of the 11 gene segments of three Thai intergenogroup reassortant strains, SKT-281, SKT-289, and LS-04, to one another. (DOCX)

S2 Table Sequence data for the 11 gene segments of nine Thai RVA strains SKT-281, SKT-289, LS-04, PCB-118, SKT-98, BD-20, NP-M51, SKT-138, and SSKT-133. (DOCX)
