# Supplementary material for: Reassortment of Human and Animal Rotavirus Gene Segments in Emerging DS-1-Like G1P[8] Rotavirus Strains
Source: PLoS One. 2016 Feb 4;11(2):e0148416. doi: 10.1371/journal.pone.0148416 (PMC4742054; doi:10.1371/journal.pone.0148416)
Supplement: S1 Table — (DOCX) [file pone.0148416.s001.docx]

**S1 Table.** Nucleotide sequence identities (%) of the 11 gene segments of three Thai intergenogroup reassortant strains, SKT-281, SKT-289, and LS-04, to one another.

| Gene | Study strain (nucleotide sequence identity) | | |
| --- | --- | --- | --- |
|  | RVA/Human-wt/THA/SKT-281/2013/G3P[8] | RVA/Human-wt/THA/SKT-289/2013/G3P[8] | RVA/Human-wt/THA/LS-04/2013/G2P[8] |
| VP7 | RVA/Human-wt/THA/SKT-289/2013/G3P[8] (100%)  RVA/Human-wt/THA/LS-04/2013/G2P[8] (73.4%) | RVA/Human-wt/THA/SKT-281/2013/G3P[8] (100%)  RVA/Human-wt/THA/LS-04/2013/G2P[8] (73.4%) | RVA/Human-wt/THA/SKT-281/2013/G3P[8] (73.4%)  RVA/Human-wt/THA/SKT-289/2013/G3P[8] (73.4%) |
| VP4 | RVA/Human-wt/THA/SKT-289/2013/G3P[8] (100%)  RVA/Human-wt/THA/LS-04/2013/G2P[8] (99.0%) | RVA/Human-wt/THA/SKT-281/2013/G3P[8] (100%)  RVA/Human-wt/THA/LS-04/2013/G2P[8] (99.0%) | RVA/Human-wt/THA/SKT-281/2013/G3P[8] (99.0%)  RVA/Human-wt/THA/SKT-289/2013/G3P[8] (99.0%) |
| VP6 | RVA/Human-wt/THA/SKT-289/2013/G3P[8] (99.9%)  RVA/Human-wt/THA/LS-04/2013/G2P[8] (98.6%) | RVA/Human-wt/THA/SKT-281/2013/G3P[8] (99.9%)  RVA/Human-wt/THA/LS-04/2013/G2P[8] (98.5%) | RVA/Human-wt/THA/SKT-281/2013/G3P[8] (98.6%)  RVA/Human-wt/THA/SKT-289/2013/G3P[8] (98.5%) |
| VP1 | RVA/Human-wt/THA/SKT-289/2013/G3P[8] (100%)  RVA/Human-wt/THA/LS-04/2013/G2P[8] (98.9%) | RVA/Human-wt/THA/SKT-281/2013/G3P[8] (100%)  RVA/Human-wt/THA/LS-04/2013/G2P[8] (98.9%) | RVA/Human-wt/THA/SKT-281/2013/G3P[8] (98.9%)  RVA/Human-wt/THA/SKT-289/2013/G3P[8] (98.9%) |
| VP2 | RVA/Human-wt/THA/SKT-289/2013/G3P[8] (100%)  RVA/Human-wt/THA/LS-04/2013/G2P[8] (99.5%) | RVA/Human-wt/THA/SKT-281/2013/G3P[8] (100%)  RVA/Human-wt/THA/LS-04/2013/G2P[8] (99.5%) | RVA/Human-wt/THA/SKT-281/2013/G3P[8] (99.5%)  RVA/Human-wt/THA/SKT-289/2013/G3P[8] (99.5%) |
| VP3 | RVA/Human-wt/THA/SKT-289/2013/G3P[8] (100%)  RVA/Human-wt/THA/LS-04/2013/G2P[8] (97.6%) | RVA/Human-wt/THA/SKT-281/2013/G3P[8] (100%)  RVA/Human-wt/THA/LS-04/2013/G2P[8] (97.6%) | RVA/Human-wt/THA/SKT-281/2013/G3P[8] (97.6%)  RVA/Human-wt/THA/SKT-289/2013/G3P[8] (97.6%) |
| NSP1 | RVA/Human-wt/THA/SKT-289/2013/G3P[8] (100%)  RVA/Human-wt/THA/LS-04/2013/G2P[8] (99.5%) | RVA/Human-wt/THA/SKT-281/2013/G3P[8] (100%)  RVA/Human-wt/THA/LS-04/2013/G2P[8] (99.5%) | RVA/Human-wt/THA/SKT-281/2013/G3P[8] (99.5%)  RVA/Human-wt/THA/SKT-289/2013/G3P[8] (99.5%) |
| NSP2 | RVA/Human-wt/THA/SKT-289/2013/G3P[8] (100%)  RVA/Human-wt/THA/LS-04/2013/G2P[8] (86.6%) | RVA/Human-wt/THA/SKT-281/2013/G3P[8] (100%)  RVA/Human-wt/THA/LS-04/2013/G2P[8] (86.6%) | RVA/Human-wt/THA/SKT-281/2013/G3P[8] (86.6%)  RVA/Human-wt/THA/SKT-289/2013/G3P[8] (86.6%) |
| NSP3 | RVA/Human-wt/THA/SKT-289/2013/G3P[8] (100%)  RVA/Human-wt/THA/LS-04/2013/G2P[8] (98.8%) | RVA/Human-wt/THA/SKT-281/2013/G3P[8] (100%)  RVA/Human-wt/THA/LS-04/2013/G2P[8] (98.8%) | RVA/Human-wt/THA/SKT-281/2013/G3P[8] (98.8%)  RVA/Human-wt/THA/SKT-289/2013/G3P[8] (98.8%) |
| NSP4 | RVA/Human-wt/THA/SKT-289/2013/G3P[8] (100%)  RVA/Human-wt/THA/LS-04/2013/G2P[8] (91.3%) | RVA/Human-wt/THA/SKT-281/2013/G3P[8] (100%)  RVA/Human-wt/THA/LS-04/2013/G2P[8] (91.3%) | RVA/Human-wt/THA/SKT-281/2013/G3P[8] (91.3%)  RVA/Human-wt/THA/SKT-289/2013/G3P[8] (91.3%) |
| NSP5 | RVA/Human-wt/THA/SKT-289/2013/G3P[8] (100%)  RVA/Human-wt/THA/LS-04/2013/G2P[8] (98.9%) | RVA/Human-wt/THA/SKT-281/2013/G3P[8] (100%)  RVA/Human-wt/THA/LS-04/2013/G2P[8] (98.9%) | RVA/Human-wt/THA/SKT-281/2013/G3P[8] (98.9%)  RVA/Human-wt/THA/SKT-289/2013/G3P[8] (98.9%) |
