# Supplementary material for: Reassortment of Human and Animal Rotavirus Gene Segments in Emerging DS-1-Like G1P[8] Rotavirus Strains
Source: PLoS One. 2016 Feb 4;11(2):e0148416. doi: 10.1371/journal.pone.0148416 (PMC4742054; doi:10.1371/journal.pone.0148416)
Supplement: S2 Table — (DOCX) [file pone.0148416.s002.docx]

**S2 Table.** Sequence data for the 11 gene segments of nine Thai RVA strains SKT-281, SKT-289, LS-04, PCB-118, SKT-98, BD-20, NP-M51, SKT-138, and SSKT-133.

| Study strain | Total reads^a^ |  | Gene | | | | | | | | | | |
| --- | --- | --- | --- | --- | --- | --- | --- | --- | --- | --- | --- | --- | --- |
|  |  |  | VP7 | VP4 | VP6 | VP1 | VP2 | VP3 | NSP1 | NSP2 | NSP3 | NSP4 | NSP5 |
| RVA/Human-wt/THA/SKT-281/2013/G3P[8] | 1,500,320 | Nucleotides; bp  (% coverage of the full-length) | 1049 (98.8%) | 2352  (99.7%) | 1355  (99.9%) | 3296  (99.8%) | 2673  (99.6%) | 2585  (99.8%) | 1565  (99.9%) | 1042  (98.4%) | 1066  (100%) | 744  (99.1%) | 792  (96.8%) |
|  |  | Deduced amino acids; aa  (% coverage of the full-length) | 326  (100%) | 775  (100%) | 397  (100%) | 1088  (100%) | 879  (100%) | 835  (100%) | 486  (100%) | 317  (100%) | 310  (100%) | 175  (100%) | 200  (100%) |
|  |  | Reads mapped to gene segment | 61,482 | 102,242 | 22,060 | 156,208 | 123,117 | 89,736 | 30,832 | 53,566 | 34,889 | 26,444 | 6,898 |
|  |  | Maximum depth of reads | 6,585 | 5,260 | 5,019 | 5,864 | 8,011 | 3,574 | 5,338 | 7,377 | 3,618 | 5,573 | 2,061 |
| RVA/Human-wt/THA/SKT-289/2013/G3P[8] | 1,594,550 | Nucleotide; bp  (% coverage of the full-length) | 1050  (98.9%) | 2350  (99.6%) | 1352  (99.7%) | 3302  (100%) | 2674  (99.6%) | 2585  (99.8%) | 1564  (99.9%) | 1045  (98.7%) | 1066  (100%) | 742  (98.8%) | 800  (97.8%) |
|  |  | Deduced amino acids; aa  (% coverage of the full-length) | 326  (100%) | 775  (100%) | 397  (100%) | 1088  (100%) | 879  (100%) | 835  (100%) | 486  (100%) | 317  (100%) | 310  (100%) | 175  (100%) | 200  (100%) |
|  |  | Reads mapped to gene segment | 62,936 | 71,850 | 23,565 | 147,705 | 119,350 | 92,518 | 36,729 | 54,327 | 46,650 | 17,966 | 6,826 |
|  |  | Maximum depth of reads | 6,361 | 4,426 | 5,733 | 5,158 | 7,814 | 3,490 | 5,340 | 7,339 | 4,370 | 3,719 | 1,698 |
| RVA/Human-wt/THA/LS-04/2013/G2P[8] | 1,706,960 | Nucleotide; bp  (% coverage of the full-length) | 1061  (99.9%) | 2358  (100%) | 1352  (99.7%) | 3292  (99.7%) | 2670  (99.5%) | 2585  (99.8%) | 1563  (99.8%) | 1047  (98.9%) | 1066  (100%) | 742  (98.8%) | 798  (97.6%) |
|  |  | Deduced amino acids; aa  (% coverage of the full-length) | 326  (100%) | 775  (100%) | 397  (100%) | 1088  (100%) | 879  (100%) | 835  (100%) | 486  (100%) | 317  (100%) | 310  (100%) | 175  (100%) | 200  (100%) |
|  |  | Reads mapped to gene segment | 62,971 | 70,413 | 40,864 | 166,301 | 134,841 | 116,054 | 48,338 | 68,558 | 66,432 | 20,919 | 14,868 |
|  |  | Maximum depth of reads | 7,062 | 5,133 | 8,496 | 5,996 | 9,122 | 4,749 | 7,519 | 7,692 | 6,245 | 6,427 | 3,958 |
| RVA/Human-wt/THA/PCB-118/2013/G1P[8] | 2,105,660 | Nucleotide; bp  (% coverage of the full-length) | 1062  (100%) | 2359  (100%) | 1355  (99.7%) | 3292  (99.7%) | 2729  (100%) | 2590  (100%) | 1562  (99.7%) | 1044  (98.6%) | 1074  (100%) | 747  (99.6%) | 652  (98.2%) |
|  |  | Deduced amino acids; aa  (% coverage of the full-length) | 326  (100%) | 775  (100%) | 397  (100%) | 1088  (100%) | 894  (100%) | 835  (100%) | 486  (100%) | 317  (100%) | 310  (100%) | 175  (100%) | 197  (100%) |
|  |  | Reads mapped to gene segment | 68,930 | 91,299 | 17,984 | 130,973 | 138,604 | 93,145 | 23,832 | 43,738 | 69,532 | 30,972 | 2,936 |
|  |  | Maximum depth of reads | 8,801 | 6,086 | 6,148 | 8,761 | 7,869 | 5,570 | 4,233 | 6,342 | 7,280 | 7,621 | 1,381 |
| RVA/Human-wt/THA/SKT-98/2013/G1P[8] | 1,685,242 | Nucleotide; bp  (% coverage of the full-length) | 1062  (100%) | 2358  (100%) | 1355  (99.7%) | 3298  (99.9%) | 2727  (99.9%) | 2591  (100%) | 1551  (99.0%) | 1047  (98.9%) | 1075  (100%) | 746  (99.5%) | 652  (98.2%) |
|  |  | Deduced amino acids; aa  (% coverage of the full-length) | 326  (100%) | 775  (100%) | 397  (100%) | 1088  (100%) | 894  (100%) | 835  (100%) | 486  (100%) | 317  (100%) | 310  (100%) | 175  (100%) | 197  (100%) |
|  |  | Reads mapped to gene segment | 71,320 | 93,620 | 21,361 | 144,191 | 157,489 | 96,327 | 25,164 | 51,157 | 72,362 | 39,846 | 5,646 |
|  |  | Maximum depth of reads | 9,503 | 5,463 | 6,704 | 9,078 | 10,471 | 6,421 | 3,876 | 6,443 | 7,546 | 9,373 | 1,934 |
| RVA/Human-wt/THA/BD-20/2013/G2P[4] | 1,293,410 | Nucleotide; bp  (% coverage of the full-length) | 1055  (99.3%) | 2349  (99.6%) | 1355  (99.9%) | 3292  (99.7%) | 2673  (99.6%) | 2591  (100%) | 1565  (99.9%) | 1047  (98.9%) | 1066  (100%) | 729  (97.1%) | 786  (96.1%) |
|  |  | Deduced amino acids; aa  (% coverage of the full-length) | 326  (100%) | 775  (100%) | 397  (100%) | 1088  (100%) | 879  (100%) | 835  (100%) | 486  (100%) | 317  (100%) | 310  (100%) | 175  (100%) | 200  (100%) |
|  |  | Reads mapped to gene segment | 53,765 | 74,517 | 20,716 | 131,274 | 101,928 | 93,279 | 53,765 | 33,937 | 64,444 | 8,764 | 3,617 |
|  |  | Maximum depth of reads | 6,752 | 5,990 | 7,197 | 5,767 | 5,303 | 4,911 | 6,752 | 7,344 | 6,599 | 4,185 | 1,228 |
| RVA/Human-wt/THA/NP-M51/2013/G2P[4] | 1,193,490 | Nucleotide; bp  (% coverage of the full-length) | 1057  (99.5%) | 2350  (99.6%) | 1353  (99.8%) | 3298  (99.9%) | 2680  (99.9%) | 2587  (99.8%) | 1565  (99.9%) | 1046  (98.8%) | 1066  (100%) | 736  (98.0%) | 794  (97.1%) |
|  |  | Deduced amino acids; aa  (% coverage of the full-length) | 326  (100%) | 775  (100%) | 397  (100%) | 1088  (100%) | 879  (100%) | 835  (100%) | 486  (100%) | 317  (100%) | 310  (100%) | 175  (100%) | 200  (100%) |
|  |  | Reads mapped to gene segment | 46,800 | 82,377 | 18,573 | 132,203 | 127,544 | 102,944 | 36,458 | 32,076 | 45,603 | 6,626 | 4,629 |
|  |  | Maximum depth of reads | 5,714 | 8,095 | 6,313 | 6,837 | 11,099 | 6,233 | 7,030 | 6,861 | 4,572 | 3,168 | 1,440 |
| RVA/Human-wt/THA/SKT-138/2013/G2P[4] | 1,743,610 | Nucleotide; bp  (% coverage of the full-length) | 1048  (98.7%) | 2347  (99.5%) | 1355  (99.9%) | 3290  (99.6%) | 2673  (99.6%) | 2588  (99.9%) | 1563  (99.8%) | 1049  (99.1%) | 1066  (100%) | 738  (98.3%) | 794  (97.1%) |
|  |  | Deduced amino acids; aa  (% coverage of the full-length) | 326  (100%) | 775  (100%) | 397  (100%) | 1088  (100%) | 879  (100%) | 835  (100%) | 486  (100%) | 317  (100%) | 310  (100%) | 175  (100%) | 200  (100%) |
|  |  | Reads mapped to gene segment | 25,293 | 31,634 | 10,028 | 40,291 | 41,977 | 35,823 | 19,544 | 31,222 | 28,842 | 6,415 | 4,471 |
|  |  | Maximum depth of reads | 3,144 | 2,372 | 3,083 | 1,836 | 2,584 | 1,859 | 2,945 | 3,539 | 2,643 | 2,471 | 1,271 |
| RVA/Human-wt/THA/SSKT-133/2013/G2P[4] | 1,454,684 | Nucleotide; bp  (% coverage of the full-length) | 1049  (98.8%) | 2347  (99.5%) | 1344  (99.1%) | 3288  (99.6%) | 2673  (99.6%) | 2583  (99.7%) | 1563  (99.8%) | 1043  (98.5%) | 1063  (99.7%) | 704  (93.7%) | 789  (96.5%) |
|  |  | Deduced amino acids; aa  (% coverage of the full-length) | 326  (100%) | 775  (100%) | 397  (100%) | 1088  (100%) | 879  (100%) | 835  (100%) | 486  (100%) | 317  (100%) | 310  (100%) | 175  (100%) | 200  (100%) |
|  |  | Reads mapped to gene segment | 6,843 | 11,873 | 2,937 | 12,575 | 15,160 | 12,315 | 6,893 | 7,968 | 7,636 | 1,419 | 1,348 |
|  |  | Maximum depth of reads | 795 | 693 | 921 | 611 | 737 | 548 | 879 | 962 | 769 | 601 | 268 |

^a^Sequence reads remaining after adapter trimming.
